# Convergent Multistage Evidence Implicates the CCR2–Artemin Immune–Inflammation Axis in Acute Myeloid Leukemia

**DOI:** 10.1155/mi/2476470

**Published:** 2026-01-31

**Authors:** Yi Jin, Hui-Min Lu, Xing-Hao Yu, Ming-Zhu Su, Jun Li, Xiao-Min Li, Jian-Hua Jin, Li-Ting Zhang, Yue Wang

**Affiliations:** ^1^ Wujin Hospital Affiliated with Jiangsu University, Changzhou, 213017, Jiangsu, China, ujs.edu.cn; ^2^ The Wujin Clinical College of Xuzhou Medical University, Changzhou, 213017, Jiangsu, China, xzmc.edu.cn; ^3^ Wujin Institute of Molecular Diagnostics and Precision Cancer Medicine of Jiangsu University, Wujin Hospital Affiliated with Jiangsu University, Changzhou, 213017, Jiangsu, China, ujs.edu.cn; ^4^ Jiangsu Province Key Laboratory of Anesthesiology, Xuzhou Medical University, Xuzhou, 221004, Jiangsu, China, xzmc.edu.cn; ^5^ Department of Outpatient and Emergency, The First Affiliated Hospital of Soochow University, Suzhou, 215123, Jiangsu, China, sdfyy.cn; ^6^ National Clinical Research Center for Hematologic Diseases, Jiangsu Institute of Hematology, The First Affiliated Hospital of Soochow University, Suzhou, 215123, Jiangsu, China, sdfyy.cn; ^7^ Jiangsu Province Key Laboratory of Tumor Biotherapy, Xuzhou Medical University, Xuzhou, 221004, Jiangsu, China, xzmc.edu.cn

**Keywords:** acute myeloid leukemia, *ARTN*, *CCR2*, genetic risk score, immune cells, mediation analysis, Mendelian randomization

## Abstract

**Background:**

The immune system and inflammatory proteins influence hematologic malignancies, but causal links with immune cell phenotypes are unclear.

**Methods:**

We applied a prespecified, multistage workflow: two‐sample and multivariable Mendelian randomization (MVMR; 731 immune traits across 12 hematologic cancers), two‐step mediation Mendelian randomization (MR) of 91 circulating inflammatory proteins, MAGMA/FUMA gene and pathway enrichment, and external validation with trait‐specific genetic risk scores (GRSs) in UK Biobank (UKB). We then performed *CCR2* perturbation assays in human monocytic leukemia cell line (THP‐1) and immortalized bone marrow‐derived macrophage (IBMDM) cells with artemin (*ARTN*) mRNA readouts and examined proteomic correlations for *ARTN* using the Olink inflammatory panel.

**Results:**

Eight immune phenotypes showed FDR–significant causal associations with malignancy, seven of which remained independent in MVMR. In acute myeloid leukemia (AML), *CCR2* on *CD62L* ^+^ myeloid dendritic cells (DCs) was associated with lower risk, whereas *BAFF-R* and *CD19* on transitional B cells were associated with higher risk, *CD19* on IgD^−^CD38^dim B cells was associated with chronic myeloid leukemia (CML), and HLA‐DR^+^ NK cells were protective in non‐Hodgkin lymphoma (NHL). Mediation MR identified three protein mediators—*CD40L*, *IL-33*, and *ARTN*, with *ARTN* mediating the *CCR2*‐AML association. GRS analyses reproduced risk directions, most prominently the protective *CCR2*–AML association. In THP‐1 and IBMDM models, *CCR2* inhibition or knockdown increased *ARTN* mRNA expression, functionally supporting a *CCR2*→*ARTN* regulatory relationship. Proteomic correlations positioned *ARTN* with immune‐metabolic proteins (*CLEC6A*, *SIGLEC6*, *NPC2*, and *MTHFD2*). Pathway analyses highlighted membrane‐proximal processes (external plasma membrane and IgG binding) and a 16p11.2 signal.

**Conclusion:**

This integrative analysis identified *CCR2*–*ARTN* as a mechanistically supported immune‐inflammation axis contributing to AML risk, offering a potential therapeutic target and warrants direct validation in primary *CD62L* ^+^ myeloid DCs.

## 1. Introduction

Hematologic malignancies comprise a diverse group of cancers arising from blood‐forming tissues of the bone marrow and lymphatic system, including acute myeloid leukemia (AML), acute lymphoblastic leukemia (ALL), chronic myeloid leukemia (CML), chronic lymphocytic leukemia (CLL), Hodgkin lymphoma (HL), non‐HL (NHL), and precursor states such as monoclonal gammopathy of undetermined significance (MGUS) and myelodysplastic syndromes (MDS) [1–6]. Despite advances in chemotherapy, targeted agents, and transplantation, marked clinical and molecular heterogeneity remains a barrier to durable cure, underscoring the need to delineate how the immune system shapes disease initiation and progression [7–9].

Observational and experimental studies suggest that specific immune‐cell phenotypes and their cytokine outputs can either promote or restrain tumor development. For example, insufficient costimulatory signaling by leukemic blasts can induce T cell tolerance in pre‐B ALL and AML [7], chronic B cell receptor (BCR) signaling drives lymphomagenesis in diffuse large B cell lymphoma (DLBCL) [10], and circulating inflammatory proteins remodel the tumor microenvironment—illustrated by IL‐6 in multiple myeloma (MM) and IL‐7 in T cell ALL (T‐ALL) [11–14]. In addition, immune checkpoint blockade is closely related to the treatment and prognosis of tumors [15, 16]. However, such evidence is vulnerable to confounding and reverse causation and does not establish whether circulating proteins mediate immune‐to‐cancer effects.

To address this gap, we prespecified a stepwise workflow: two‐sample Mendelian randomization (MR) [17, 18] to infer causal effects of immune traits on hematologic malignancies, multivariable MR (MVMR) to disentangle correlated immune exposures, mediation MR across 91 circulating inflammatory proteins to test indirect effects (IDEs), MAGMA–based gene and pathway annotation to enhance biological interpretation, external validation of trait‐specific genetic risk scores (GRSs) in the UK Biobank (UKB) [19], and targeted cell‐based assays. We screened 731 immune phenotypes across 12 hematologic malignancies and applied a tiered prioritization scheme that triangulated signals across these analytical layers. Convergent evidence highlighted a *CCR2–*artemin (*ARTN*) immune–inflammation axis in AML, which we subsequently interrogated experimentally by perturbing *CCR2* and quantifying *ARTN* responses. This broad‐to‐precise workflow connects population genetics, cohort‐level replication, and mechanistic experimentation to nominate tractable immune mediators of hematologic malignancies.

## 2. Materials and Methods

### 2.1. Study Design

**Figure 1:**
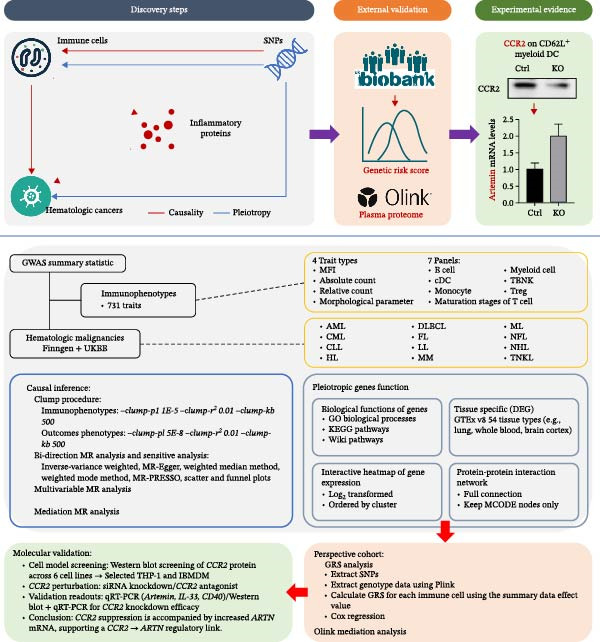
Study design and methodology for MR analysis of immune cell phenotypes and hematologic malignancies.

This study followed a prespecified, stepwise workflow (Figure 1) to link immune‐cell phenotypes with hematologic malignancies and to move from discovery to biological prioritization. First, we performed two‐sample MR to screen for causal effects of immune traits across 12 hematologic malignancies. Second, for outcomes influenced by multiple immune traits, we applied MVMR to estimate independent effects. Third, we evaluated indirect pathways via mediation MR across 91 circulating inflammatory proteins. Fourth, we used MAGMA–based gene and pathway analyses to contextualize implicated biology. Fifth, we conducted external validation by testing trait‐specific GRSs in the UKB. Finally, guided by convergent evidence, we performed targeted cell‐based assays to probe a prioritized *CCR2*–*ARTN* axis in AML. This broad‐to‐precise design ensures that statistical signals are triangulated across orthogonal layers before experimental interrogation.

### 2.2. Data Sets

We obtained summary statistics for 731 immune phenotypes from the GWAS Catalog (ID range GCST0001391–GCST0002121) [20]. These traits included absolute cell counts (AC, *n* = 118), relative cell counts (RC, *n* = 192), median fluorescence intensities (MFI, *n* = 389) reflecting surface antigen levels, and morphometric parameters (MP, *n* = 32), spanning B cells, dendritic cells (DCs), T cells at distinct maturation stages, monocytes, myeloid cells, TBNK panels, and Treg panels. The underlying GWASs were conducted in 3757 participants of European ancestry with adjustment for sex, age, and age [2]; genotypes (~22 million single nucleotide polymorphisms [SNPs]) were assayed on high‐density arrays and imputed to a European reference panel.

Summary statistics for 12 hematologic malignancy endpoints were drawn from a meta‐analysis of UKB and FinnGen (Release 10) [21] (https://public-metaresults-fg-ukbb.finngen.fi/), with case definitions and sample sizes summarized in Table 1.

**Table 1:** Summary statistics for 12 lymphoma subtypes from UK Biobank and FinnGen (R10) databases.

Subtype	FinnGen cases	FinnGen controls	UK Biobank cases	UK Biobank controls
Acute myeloid leukemia (AML)	244	314,192	409	414,840
Chronic myeloid leukemia (CML)	115	314,192	171	414,840
Chronic lymphocytic leukemia (CLL)	668	314,189	650	414,840
Diffuse large B cell lymphoma (DLBCL)	1050	314,193	761	414,840
Follicular lymphoma (FL)	1181	324,650	536	414,840
Hodgkin lymphoma (HL)	846	324,650	317	414,840
Lymphoid leukemia (LL)	1617	324,650	802	414,840
Multiple myeloma (MM)	1337	324,650	760	419,771
Myeloid leukemia (ML)	734	324,650	567	419,964
Nonfollicular lymphoma (NFL)	2811	324,650	1092	419,439
Non‐Hodgkin lymphoma (NHL)	1171	324,650	1481	419,050
T/NK cell lymphoma (TNKL)	363	324,650	151	420,380

Plasma proteomics for 91 circulating inflammatory proteins were taken from a large pQTL meta‐analysis of 14,824 European‐ancestry participants measured on the Olink Target Inflammation panel, with linear‐regression GWASs meta‐analyzed across 11 cohorts. Seventeen genome‐wide significant associations (*p* ≤ 5 × 10^−10^) mapped 108 loci to 70 proteins and were replicated in independent datasets (e.g., ARISTOTLE and deCODE) [22].

Across all datasets, we applied uniform SNP–level quality control: removal of nonbiallelic variants, ambiguous A/T or C/G alleles without strand resolution, missing rsIDs, duplicate rsIDs or base‐pair positions, variants absent from 1000 Genomes Phase 3, allele mismatches relative to 1000 Genomes Phase 3, imputation INFO < 0.9, and variants on chromosomes X and Y.

### 2.3. Instrumental Variable (IV) Selection

Following previous studies [20, 23, 24], for each immune trait, we selected instruments at *p*  < 1 × 10^−5^ and performed LD clumping using PLINK (1000 Genomes EUR reference; *r* ^2^ < 0.001; 500 kb window). Palindromic SNPs with intermediate allele frequencies were excluded unless strand could be resolved. When an instrument was missing in an outcome dataset, a high‐LD proxy (*r* ^2^ ≥ 0.80, EUR) was substituted where available. We harmonized effect alleles across exposure, mediator, and outcome datasets. For each trait, we estimated the variance explained (*R* ^2^) and the first‐stage F‐statistic to assess instrument strength, retaining sets with *F* > 10 to limit weak‐instrument bias.

### 2.4. MR Analysis

We used inverse‐variance weighted (IVW) MR as the primary estimator of the causal effect of immune traits on hematologic malignancies, under standard instrumental‐variable assumptions. Multiple testing was controlled using the false discovery rate (FDR *q* < 0.05) across trait–outcome tests. Analyses were conducted in R (v3.5.3) using the MendelianRandomization package for core estimators and MR‐PRESSO for outlier detection; additional sensitivity estimators are detailed below. This study used only publicly available, deidentified summary statistics and did not involve direct human or animal experimentation.

Several sensitivity analyses were performed to ensure the robustness of our findings. First, we used the Q test for heterogeneity within the IVW and MR‐Egger methods to detect potential violations of the MR assumptions based on the variability between the IVs [25]. Second, MR‐Egger regression was used to estimate horizontal pleiotropy through its intercept, ensuring that genetic variants were not related to both the exposure and outcome independently. Additional robustness was achieved by conducting analyses using methods with different modeling assumptions and strengths, including the weighted median and weighted mode approaches [26]. Third, we employed MR‐PRESSO to identify outliers and correct for horizontal pleiotropy [27]. Finally, single SNP analysis and leave‐one‐out (LOO) analysis were conducted to assess the influence of individual SNPs on the observed associations, ensuring that the results were not driven by any single variant. To further mitigate the influence of horizontal pleiotropy, we annotated all instrumental SNPs against the NHGRI‐EBI GWAS Catalog (v1.0, release 2025‐04‐14). SNPs that were previously reported to be significantly associated (*p* < 5 × 10^−8^) with traits biologically related to the outcome of interest were considered potentially pleiotropic and were excluded prior to MR estimation (Supporting Information 2: Table S1). Trait relevance was determined based on disease ontology and manual curation of related phenotypes. This filtering step was performed for each significant exposure–outcome pair separately.

### 2.5. MVMR and Mediation Analysis

For outcomes with multiple immune traits showing evidence of causality in univariable MR, we performed MVMR using IVW to estimate mutually adjusted effects and to evaluate whether each trait retained an independent association with risk. Instruments were constructed from the union of trait‐specific variants, clumped to independence (*r* ^2^ < 0.001), harmonized across all datasets, and restricted to SNPs present in all relevant exposure and outcome summary statistics. We examined instrument strength using conditional F‐statistics and assessed residual pleiotropy via MVMR heterogeneity metrics.

To test whether circulating inflammatory proteins mediate immune‐to‐cancer effects, we implemented two‐step mediation MR using summary statistics [17, 28, 29]. In step 1, we estimated the causal effect of each prioritized immune trait on protein levels. In step 2, we estimated the effect of the protein on the malignancy while adjusting for the immune trait using MVMR. The IDE was computed as the product of coefficients (*a* × *b*); 95% confidence intervals (CIs) and *p* values were derived from bootstrap resampling (10,000 draws). We also report the proportion mediated where appropriate. Multiple testing across proteins was controlled by FDR. This framework quantifies whether circulating inflammatory proteins plausibly lie on the pathway from immune traits to malignancy risk.

### 2.6. Biological Role and Pathway Analysis of Causal Immune Cells

To further investigate the biological roles of immune cells in the development of complex phenotypes and to obtain comprehensive and reliable pathway analysis results, we conducted a Generalized Gene‐Set Analysis of GWAS Data (MAGMA) on the eight identified causal immune cell types [30]. This analysis aimed to identify candidate pleiotropic genes, with significance set at *p*  < 0.05/*N*_genes_ = 3 × 10^−6^. Subsequently, we performed tissue‐specific and pathway analyses on the mapped key genes. Functional mapping and annotation (FUMA) of Genome‐Wide Association Studies was employed to determine the biological functions of pleiotropic loci [31]. Tissue‐specific expression and enrichment analyses were conducted using FUMA (https://fuma.ctglab.nl/). Additionally, we utilized the Metascape tool to integrate Gene Ontology (GO), Kyoto Encyclopedia of Genes and Genomes (KEGG), WikiPathways (WP), and cell‐specific gene set databases for comprehensive pathway analysis.

### 2.7. External Validation in UKB

To externally validate the biological relevance of MR‐identified immune traits, we constructed GRSs for eight immune cell phenotypes using lead SNPs derived from immunophenotyping GWAS. SNPs were selected based on clumping thresholds (*p* < 1 × 10^−5^ and *r^2^* < 0.001) and harmonized by effect allele orientation. The *β* coefficients (effect sizes) from the immune cell GWAS were used as weights in GRS calculation to ensure that the scores represent genetically predicted immune cell traits. Individual‐level genotype data were extracted from the UKB cohort (Application ID: 41542) using PLINK 2.0. Trait‐specific GRSs were computed as the weighted sum of allele dosages multiplied by corresponding GWAS effect sizes:

GRSi=∑j=1nβj×allele dosageij.

The resulting GRSs were standardized to *Z*‐scores before downstream analyses. We then assessed associations between each GRS and incident hematologic malignancies—including AML, CML, HL, and NHL—based on linked ICD‐10 and ICD‐9 codes from the UKB cancer registry. Time‐to‐event was defined from baseline to first diagnosis or censoring, and Cox proportional hazards models were used to estimate hazard ratios (HRs) and 95% confidence intervals, adjusting for age, sex, genotyping batch, Townsend deprivation index (TDI), type 2 diabetes, osteoporosis status, and baseline blood cell counts. The analysis was restricted to individuals of genetically inferred European ancestry.

We next evaluated circulating plasma proteins as mediators of immune trait–associated risk using UKB Olink assays. Proteins were rank‐normalized, standardized, and tested in Cox models adjusted for demographic, socioeconomic, and hematologic covariates. We applied a counterfactual mediation framework to quantify direct and IDEs per 1 SD change in exposure, with 1000 bootstrap iterations. Annotation of Olink identifiers enabled mapping to gene symbols. Proteins with significant IDEs after FDR correction were interpreted as candidate mediators.

### 2.8. Functional Validation of *CCR2*–Mediated Regulation of *ARTN* Expression

Our assays tested whether *CCR2* perturbation directionally modulates *ARTN* transcription in myeloid systems with robust *CCR2* expression. Human monocytic leukemia cell line (THP‐1) and immortalized bone marrow‐derived macrophage (IBMDM; murine macrophage) were selected for assayability and stable *CCR2* levels. These models are not intended to represent *CD62L* ^+^ mDCs; rather, they provide monocyte/macrophage contexts to probe the *CCR2*→*ARTN* modulation predicted by the MR/mediation analyses.

#### 2.8.1. Cell Line Screening and Culture Conditions

Six commonly used cell lines (THP‐1, 293 T, HeLa, A549, IBMDM, and L929) were cultured in RPMI‐1640 (for THP‐1) or DMEM (for the others) medium (Gibco, Shanghai, China), each supplemented with 10% fetal bovine serum (FBS; Gibco, USA) and 1% penicillin–streptomycin, and maintained at 37°C in a humidified incubator with 5% CO_2_. To identify suitable models, *CCR2* protein expression was assessed by Western blotting in each cell line, with three independent biological replicates. Relative expression was quantified using ImageJ and normalized to GAPDH. THP‐1 and IBMDM cells, which consistently exhibited higher *CCR2* protein levels than other tested lines, were selected for subsequent validation.

#### 2.8.2. *CCR2* Inhibition and Knockdown Experiments

To assess the effect of *CCR2* suppression on downstream gene expression, two strategies were employed: (1) Pharmacological inhibition: Cells were treated with 10 *μ*M *CCR2* antagonist 4 hydrochloride (MedChemExpress, HY‐103362, 10 mM stock in DMSO) for 24 h. Vehicle control (0.1% DMSO) was used in parallel. (2) Small interfering RNA (siRNA)–mediated knockdown: Cells were transfected with 50 nM *CCR2*–targeting siRNA (GenePharma, Suzhou, China) using Lipofectamine RNAiMAX (Invitrogen, USA) in Opti‐MEM medium (Thermo Fisher, USA). A nontargeting siRNA served as the negative control, while two independent *CCR2*–targeting siRNAs were used to assess knockdown effects. After 6 h, media were replaced with complete medium, and cells were harvested after 48 h. To improve reliability, two independent siRNA sequences were tested, and transfection efficiency was confirmed by assessing *CCR2* mRNA knockdown (≥70%) via quantitative real‐time polymerase chain reaction (qRT‐PCR).

#### 2.8.3. RNA Extraction and qRT‐PCR

Total RNA was extracted using TRIzol (Invitrogen, USA), and reverse transcription was performed using the HiScript III RT Kit (Vazyme, Nanjing, China). Quantitative real‐time PCR was conducted with SYBR Green Master Mix (Vazyme, China) on a QuantStudio 5 system (Applied Biosystems, USA). Primers were designed based on NCBI reference sequences via Primer‐BLAST and synthesized by Sangon Biotech (Shanghai, China). ACTB and GAPDH were used as dual reference genes. The 2^-^ *ΔΔ*Ct method was used to calculate relative expression levels.

#### 2.8.4. Western Blotting

Proteins were extracted using RIPA buffer (Beyotime, China) with protease inhibitor cocktail (Roche, Switzerland). Concentrations were determined via BCA assay (Beyotime). Equal protein amounts were separated via SDS‐PAGE, transferred to polyvinylidene difluoride (PVDF) membranes (Millipore, USA), and probed with the following primary antibodies overnight at 4°C: anti‐*CCR2* (Rabbit mAb, ABclonal Technology, A2385, 1:1000) and anti‐GAPDH (CST, #4970, 1:5000). After incubation with HRP–conjugated secondary antibody (ABclonal Technology, 1:5000), signals were detected using enhanced chemiluminescence (ECL) (Thermo Fisher, USA) and quantified by ImageJ. Molecular weight markers were included, and exposure times were optimized to avoid saturation. *ARTN*, *IL-33*, and *CD40* were measured at the transcript level.

#### 2.8.5. Experimental Design and Statistical Analysis

Downstream readouts (*ARTN*, *IL-33*, and *CD40*) were quantified at the mRNA level. For *CCR2*, both protein (WB) and mRNA (qRT‐PCR) were measured to screen models and confirm knockdown. All experiments were performed with ≥3 independent biological replicates. Statistical analyses were conducted using GraphPad Prism 9.0 (GraphPad Software, USA). Two‐group comparisons were analyzed using two‐tailed unpaired Student’s *t*‐test. Data are presented as mean ± standard deviation (SD), and *p*  < 0.05 was considered statistically significant.

## 3. Results

### 3.1. Causal Associations of Immune‐Cell Phenotypes With Hematologic Malignancies

**Figure 2:**
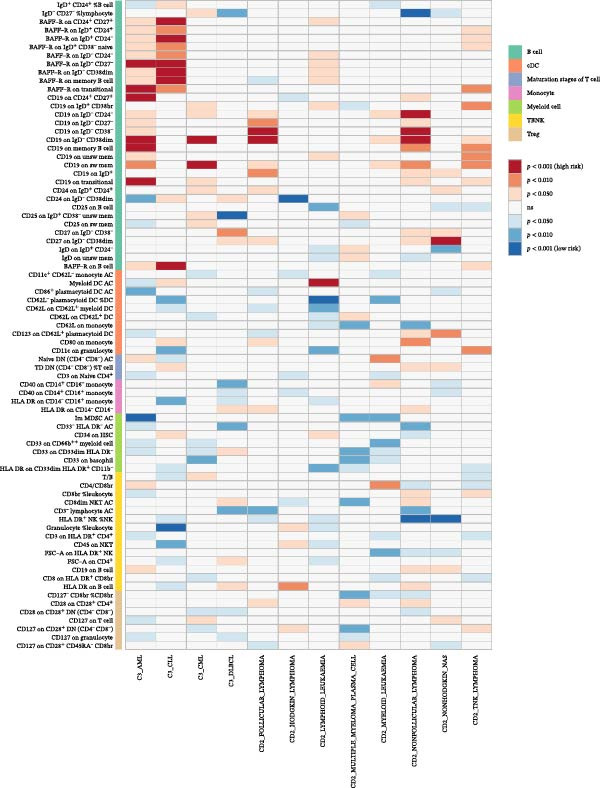
Immune cell phenotypes significantly associated with three or more hematologic malignancies before FDR correction. This figure shows immune cell phenotypes significantly associated (*p*  < 0.05) with three or more hematologic malignancy subtypes without FDR multiple corrections. The color coding indicates the type of immune cell and the strength of the association, with red shades indicating high risk and blue shades indicating low risk.

Across 731 immune phenotypes and 12 hematologic malignancies, two‐sample MR identified eight immune traits meeting the FDR threshold (Supporting Information 2: Tables S2–S4), with summary effects visualized in Figure 2. In AML, *BAFF-R* on transitional B cells (OR 1.09, 95% CI 1.04–1.15; *p*  < 0.001) and *CD19* on transitional B cells (OR 1.25, 95% CI 1.13–1.39; *p*  < 0.001) were positively associated with risk, whereas *CCR2* on *CD62L* ^+^ myeloid DCs was inversely associated (OR 0.82, 95% CI 0.74–0.91; *p*  < 0.001). In HL, the IgD^-^ CD27^-^ absolute count (AC) was protective (OR 0.73, 95% CI 0.64–0.84; *p*  < 0.001), while IgD^-^ CD38br %B cells conferred higher risk (OR 1.36, 95% CI 1.16–1.59; *p*  < 0.001); CD24 on IgD^-^ CD38dim B cells showed an inverse association (OR 0.92, 95% CI 0.89–0.96; *p*  < 0.001). In CML, *CD19* on IgD^-^ CD38dim B cells was positively associated (OR 1.37, 95% CI 1.18–1.59; *p*  < 0.001). For NHL, HLA‐DR^+^ NK cells were protective (OR 0.90, 95% CI 0.86–0.95; *p*  < 0.001). Method‐level concordance for the eight prioritized pairs is shown in Figure 3A. Full MR estimates and sensitivity statistics are provided in Supporting Information 2: Tables S3–S5. IVW and MR‐Egger Q tests indicated acceptable heterogeneity; MR‐Egger intercepts showed no strong evidence of directional pleiotropy; weighted median/mode and MR‐PRESSO gave concordant directions (Supporting Information 2: Table S5). Excluding instruments flagged by our preanalysis GWAS–catalog screen yielded similar effect sizes with reduced heterogeneity (Supporting Information 2: Table S6). Reverse MR analyses were null (Supporting Information 2: Table S7). Scatter and LOO plots are shown in Supporting Information 3: Figures S1,S2.

Figure 3Causal effects of immune‐cell phenotypes on hematologic malignancies. (A) Forest plots for eight significant associations between immune cell phenotypes and hematologic malignancies identified in the MR analysis. Each plot presents the odds ratio (OR) and 95% confidence interval (CI) for the association between a specific immune cell phenotype and a hematologic malignancy across various MR methods, including IVW fixed, IVW random, D‐IVW, MR‐RAPS, weighted mode, weighted median, MR‐Egger, and MR‐PRESSO. Significant associations (*p*  < 0.05) are highlighted. (B) Results of multivariable Mendelian randomization (MVMR) analysis for significant causal relationships between immune cell phenotypes and hematologic malignancies. This figure presents the MVMR analyses conducted to account for potential confounding interactions when two or more immune cell phenotypes showed a significant causal relationship with a single hematologic malignancy.

**Figure:**
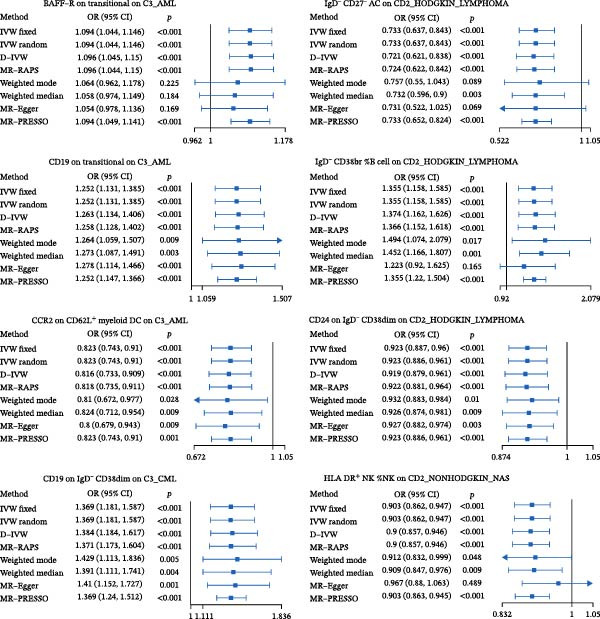
(A)

**Figure:**
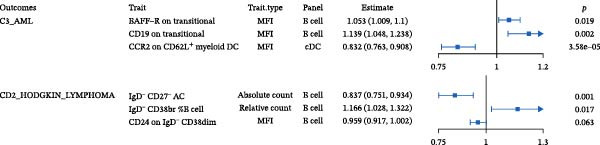
(B)

Where multiple immune traits mapped to the same malignancy, MVMR retained independent effects (Figure 3B). In AML, *BAFF-R* on transitional B cells (OR 1.05, 95% CI 1.01–1.10; *p* = 0.019), *CD19* on transitional B cells (OR 1.14, 95% CI 1.05–1.24; *p* = 0.002), and *CCR2* on *CD62L* ^+^ myeloid DCs (OR 0.83, 95% CI 0.76–0.91; *p* = 3.58 × 10^−5^) each remained significant after mutual adjustment. In HL, IgD^-^ CD27^-^ AC (OR 0.84, 95% CI 0.75–0.93; *p* = 0.001) and IgD^-^ CD38br %B cells (OR 1.17, 95% CI 1.03–1.32; *p* = 0.017) retained independent associations, whereas the *CD24* signal attenuated (OR 0.96, 95% CI 0.92–1.00; *p* = 0.063).

### 3.2. Mediation by Circulating Inflammatory Proteins

Among the eight significant immune–malignancy associations identified by MR, mediation analysis of 91 circulating inflammatory proteins revealed *ARTN* as a key downstream effector linking immune‐cell signaling to AML risk (Supporting Information 2: Table S8). For AML, *CCR2* on *CD62L* ^+^ myeloid DCs showed a protective IDE via *ARTN* levels (IDE = −0.0082, 95% CI −0.021 to −0.001, *p* = 0.026; total effect = −0.2128, *p*  < 0.001; direct effect = −0.2086, *p*  < 0.001), whereas *CD19* on transitional B cells conferred a risk‐enhancing IDE through *ARTN* (IDE = 0.0090, 95% CI 0.001–0.021, *p* = 0.030; total effect = 0.1697, *p* = 0.001; direct effect = 0.1734, *p*  < 0.001). These findings indicate that *ARTN* can transmit effects in both innate and adaptive contexts, without implying a shared downstream program. Beyond AML, two secondary mediation routes were detected in HL. IgD^-^CD27^-^ atypical B cells showed a protective IDE via *CD40L* receptor levels (IDE = −0.0560, 95% CI −0.1117 to −0.0112, *p* = 0.001), and CD24 on IgD^-^CD38^dim B cells showed a protective IDE via *IL-33* (IDE = −0.0067, 95% CI −0.0150 to −0.001, *p* = 0.001).

To extend these mediation findings at the proteomic level, we examined correlations of *ARTN* with other inflammatory proteins in the UKB cohort. In the *ARTN*–AML mediation screen, four proteins remained significant after FDR correction, with *CLEC6A* and *SIGLEC6* mediating positive IDEs and *NPC2* and *MTHFD2* showing inverse mediation patterns. *ARTN* levels correlated strongly with these four mediators (Figure 4A–D), which are involved in antigen presentation, macrophage activation, and mitochondrial metabolism. The individual IDEs (IDE1 and IDE2) are illustrated in Figure 4E, while the overall IDEs are summarized in the forest plot shown in Figure 4F. Together, these findings support *ARTN* as a core node of the *CCR2-ARTN* immune–metabolic axis and identify potential downstream effectors contributing to AML susceptibility.

Figure 4Proteomic extension of the *CCR2*–*ARTN* axis and cohort‐level genetic validation. (A–D) Correlations between *ARTN* and the four inflammatory proteins that remained significant after FDR correction in UK Biobank proteomics. (E) Path diagram summarizing two‐step mediation from *ARTN* to AML through each protein; values on arrows denote path coefficients (*β*, 95% CI). CLEC6A and SIGLEC6 show positive mediation, whereas NPC2 and MTHFD2 show inverse mediation. (F) Forest plot of the overall indirect effects (IDE; *β* with 95% CI) of the four *ARTN*–linked proteins on AML; FDR–significant mediators are highlighted. (G) Forest plot showing associations between GRSs of immune cell phenotypes and hematologic malignancies in the UK Biobank cohort. *CCR2* on *CD62L* ^+^ myeloid DCs was inversely associated with AML risk, while *CD19* on transitional B cells showed a positive association. (H–J) GRS distribution plots for three representative immune traits.

**Figure:**
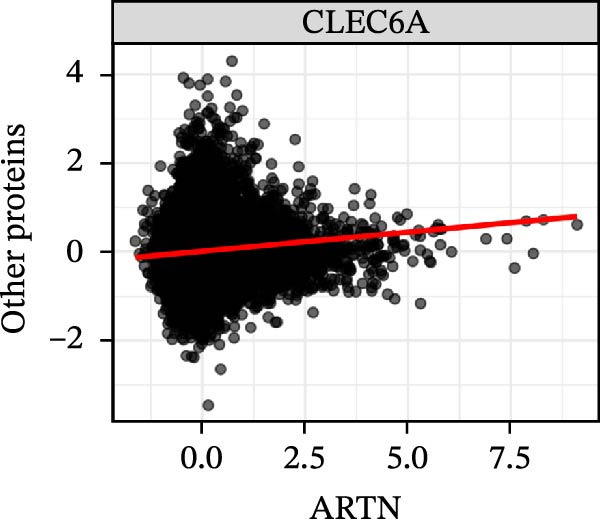
(A)

**Figure:**
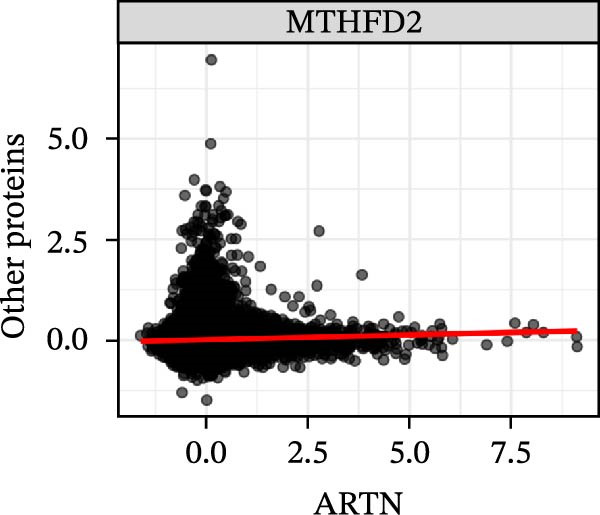
(B)

**Figure:**
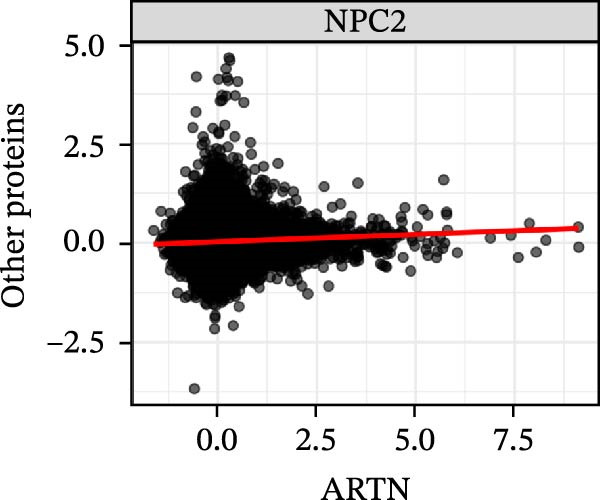
(C)

**Figure:**
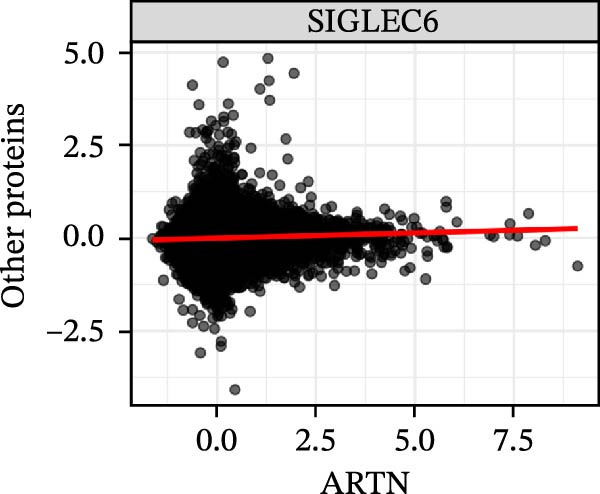
(D)

**Figure:**
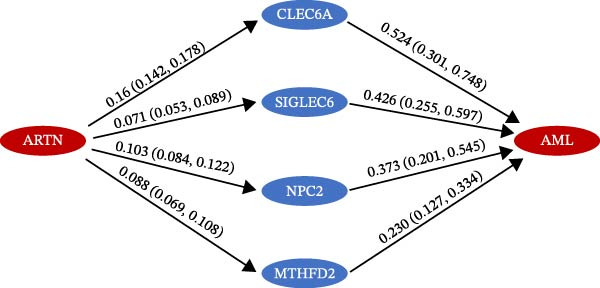
(E)

**Figure:**
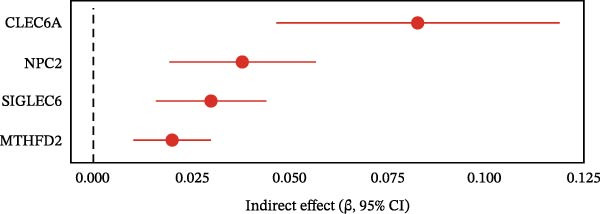
(F)

**Figure:**
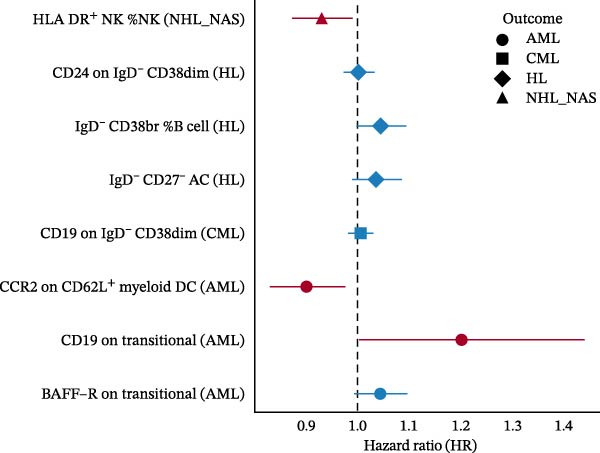
(G)

**Figure:**
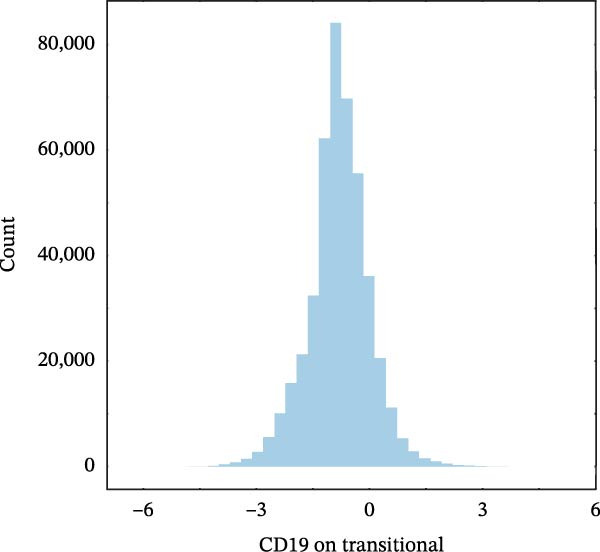
(H)

**Figure:**
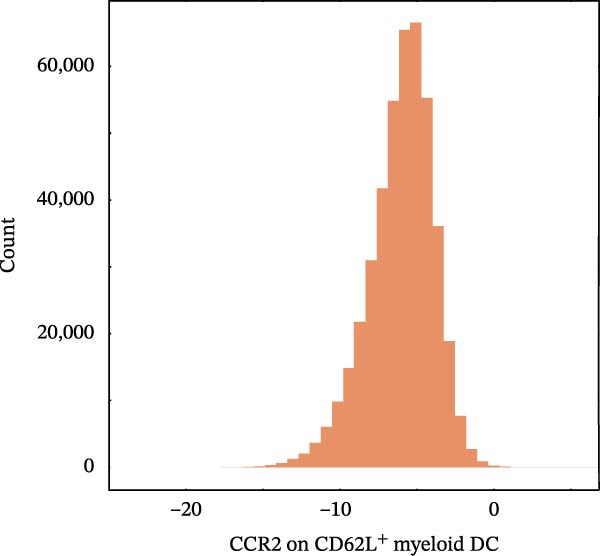
(I)

**Figure:**
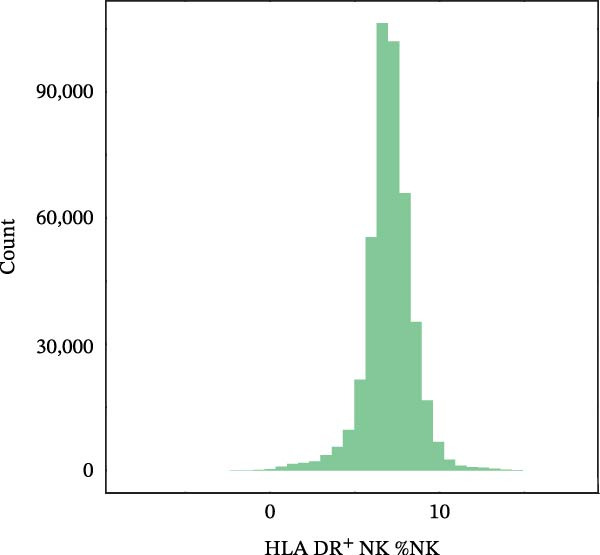
(J)

### 3.3. External Validation in a Large Prospective Cohort

To validate the causal architecture suggested by MR and mediation analyses, trait‐specific GRSs were constructed in the UKB cohort. The results consistently reproduced the predicted directions (Figure 4G). For AML, the GRS representing *CD19* expression on transitional B cells was associated with a higher ri sk (HR = 1.201, 95% CI 1.004–1.438, *p* = 0.046), whereas the GRS representing *CCR2* expression on *CD62L* ^+^ myeloid DCs was associated with a lower risk (HR = 0.901, 95% CI 0.832–0.975, *p* = 0.010). This reciprocal pattern mirrors the *CCR2-ARTN* regulatory axis identified in the mediation analysis, supporting its robustness at the cohort level. For NHL, a protective association was observed for the GRS of HLA‐DR^+^ NK cells (HR = 0.930, 95% CI 0.875–0.989, *p* = 0.020). GRS distributions for each trait are presented in Figure 4H–J.

### 3.4. Experimental Verification of the *CCR2–ARTN* Regulatory Axis

Guided by convergent genetic and cohort evidence, we tested whether perturbing *CCR2* modulates *ARTN* in myeloid monocyte/macrophage models (THP‐1, IBMDM) where *CCR2* is robustly expressed to assess directional regulation (Figure 5A,B). In IBMDM, two independent *CCR2* siRNA (KO‐1/KO‐2) reduced *CCR2* protein (Figure 5C) and *CCR2* mRNA (Figure 5D), with a concordant increase in *ARTN* mRNA expression (Figure 5E). In THP‐1, *CCR2* knockdown similarly reduced *CCR2* protein (Figure 5F) and mRNA (Figure 5G) and elevated *ARTN* mRNA (Figure 5H). Pharmacologic CCR2 antagonism decreased *CCR2* protein and mRNA in THP‐1 (Figure 5I,J) and IBMDM (Figure 5L,M), accompanied by higher *ARTN* mRNA (Figure 5K,N). *IL-33* and *CD40* mRNA also decreased after *CCR2* suppression (Supporting Information 3: Figure S3). Raw qPCR Cq values are reported in Supporting Information 2: Table S9. These assays further delineate a negative *CCR2-ARTN* regulatory relationship in myeloid models.

Figure 5Experimental validation of the *CCR2*–*ARTN* regulatory axis. (A) *CCR2* protein (Western blot) across six cell lines. (B) Densitometric quantification of *CCR2* normalized to GAPDH and expressed relative to THP‐1. IBMDM, *CCR2* siRNA: (C) Western blot showing effective reduction of *CCR2* protein after two independent siRNAs. (D) qRT‐PCR confirmation of *CCR2* mRNA knockdown. (E) *ARTN* mRNA measured by qRT‐PCR shows a concordant increase following *CCR2* silencing, consistent across both siRNAs. THP‐1, *CCR2* siRNA: (F) Western blot of *CCR2* protein demonstrating knockdown efficiency. (G) qRT‐PCR verification of reduced *CCR2* mRNA levels. (H) Elevated *ARTN* mRNA after *CCR2* knockdown in THP‐1 cells. THP‐1, pharmacologic *CCR2* inhibition: (I) Western blot showing decreased *CCR2* protein. (J) Corresponding decrease in *CCR2* mRNA and (K) increase in *ARTN* mRNA, assessed by qRT‐PCR. IBMDM, pharmacologic inhibition: (L) Western blot confirming reduced *CCR2* protein upon inhibitor treatment. (M) Consistent decrease in *CCR2* mRNA and (N) upregulation of *ARTN* mRNA detected by qRT‐PCR. For all blots, GAPDH served as loading control; *CCR2* band intensities were normalized to GAPDH. *ARTN* expression was measured by qRT‐PCR and normalized to reference genes. Bars show mean ± SD from ≥3 independent experiments; two‐tailed unpaired *t*‐tests were used.

**Figure:**
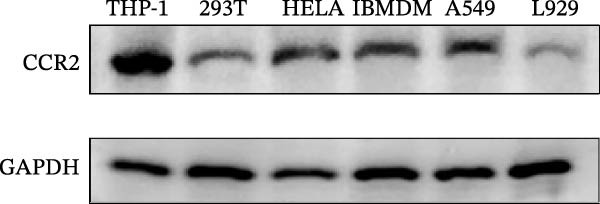
(A)

**Figure:**
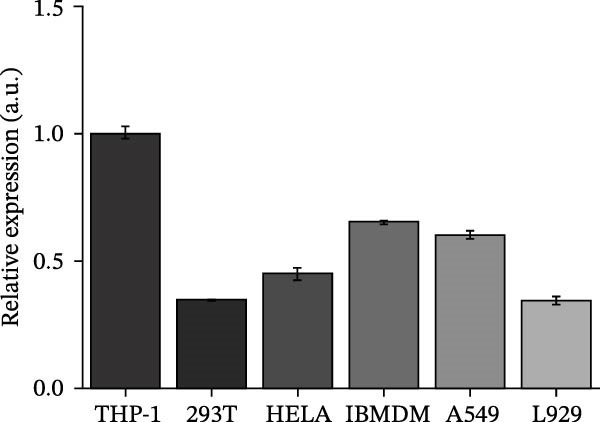
(B)

**Figure:**
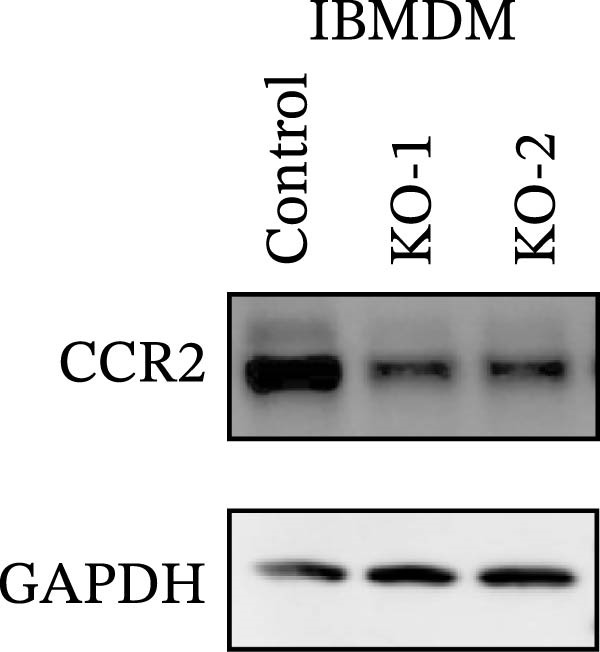
(C)

**Figure:**
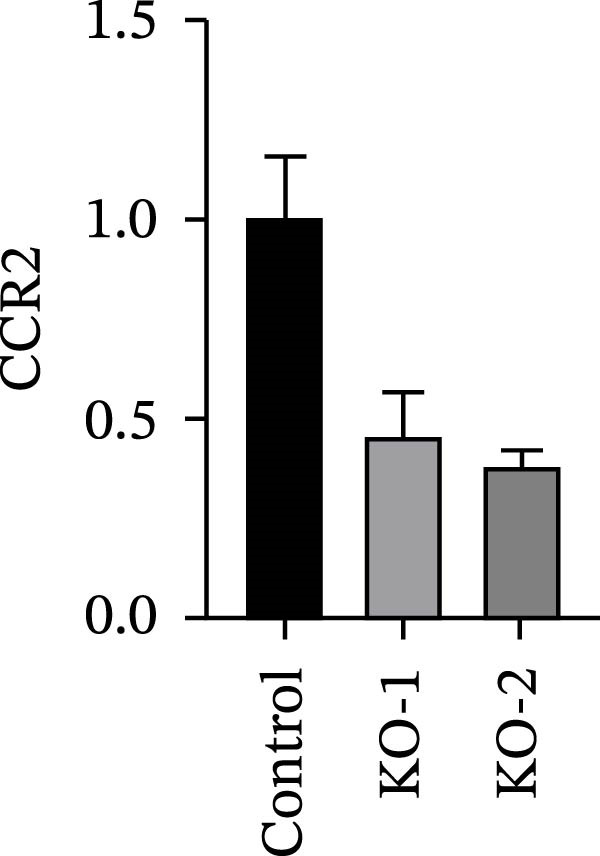
(D)

**Figure:**
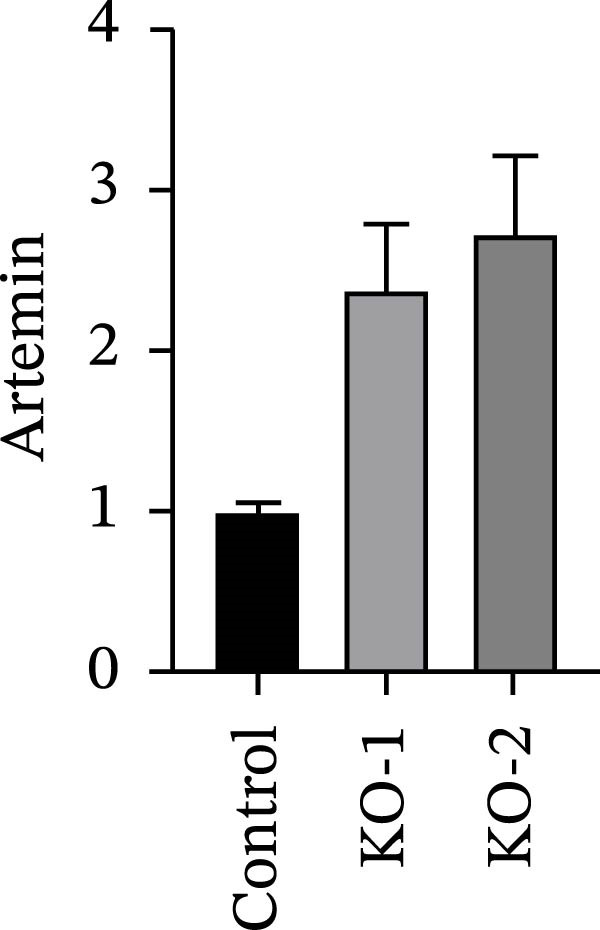
(E)

**Figure:**
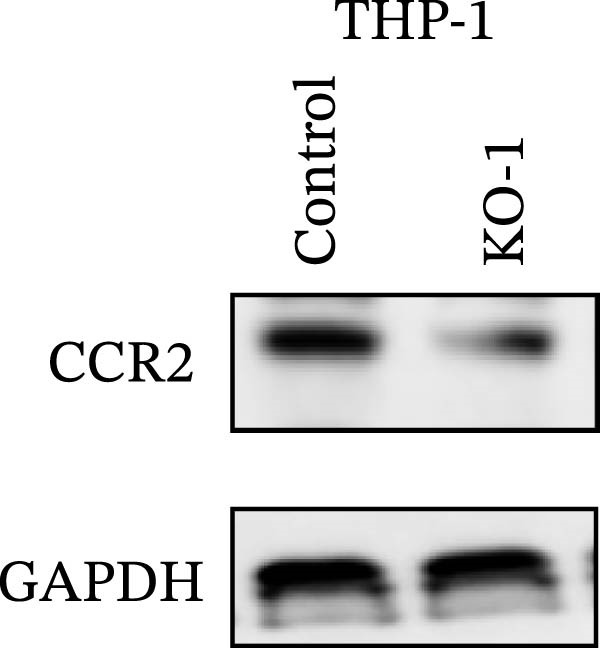
(F)

**Figure:**
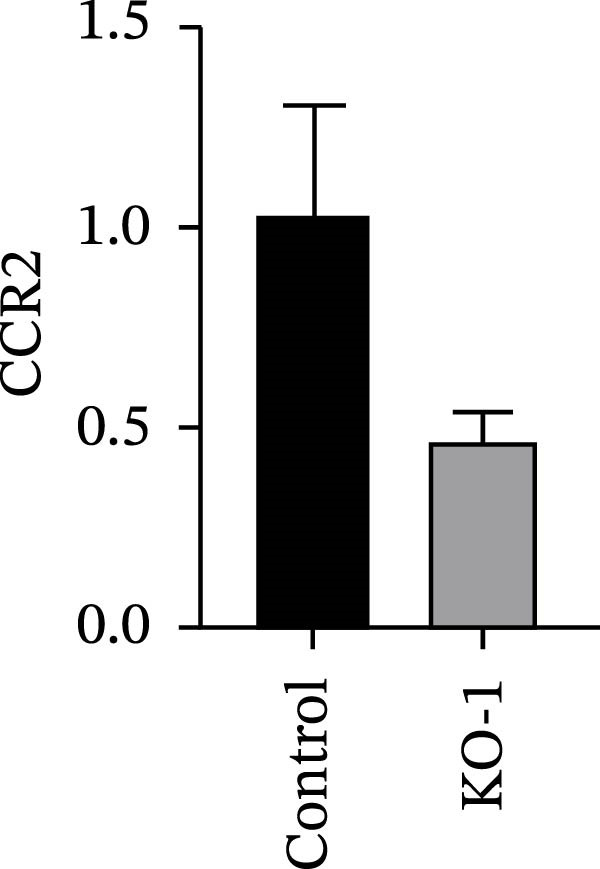
(G)

**Figure:**
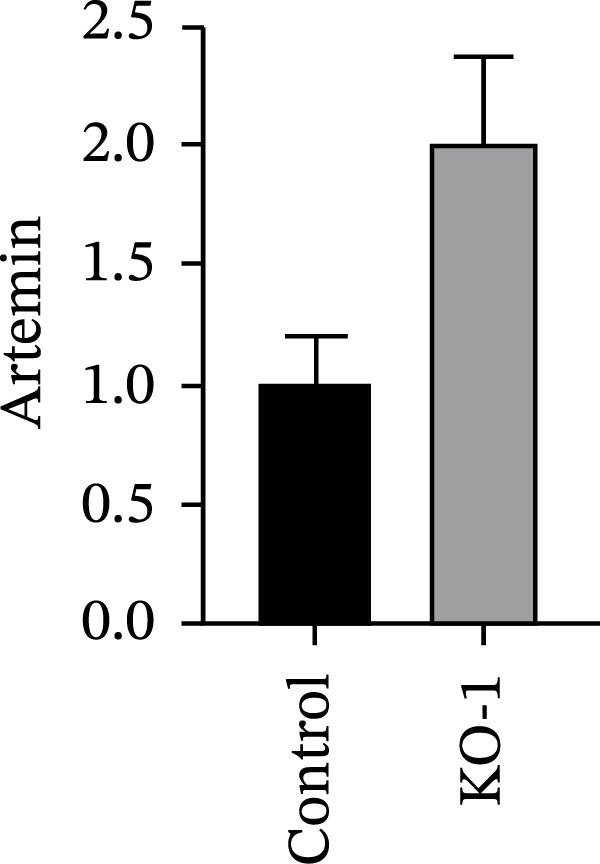
(H)

**Figure:**
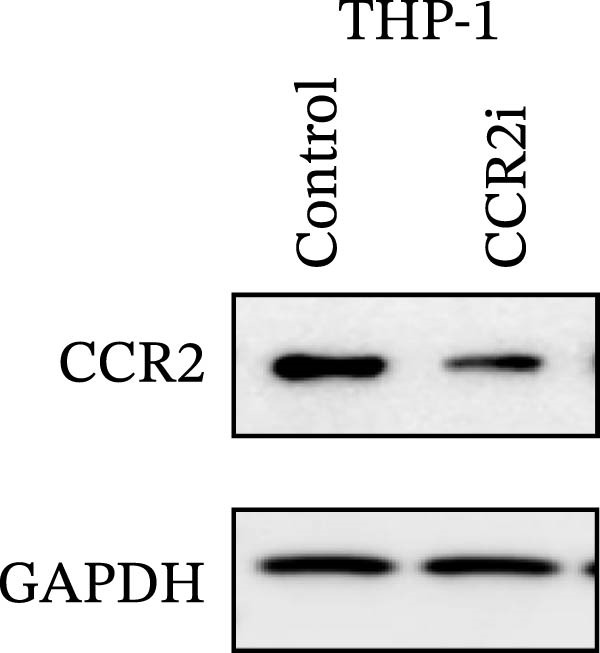
(I)

**Figure:**
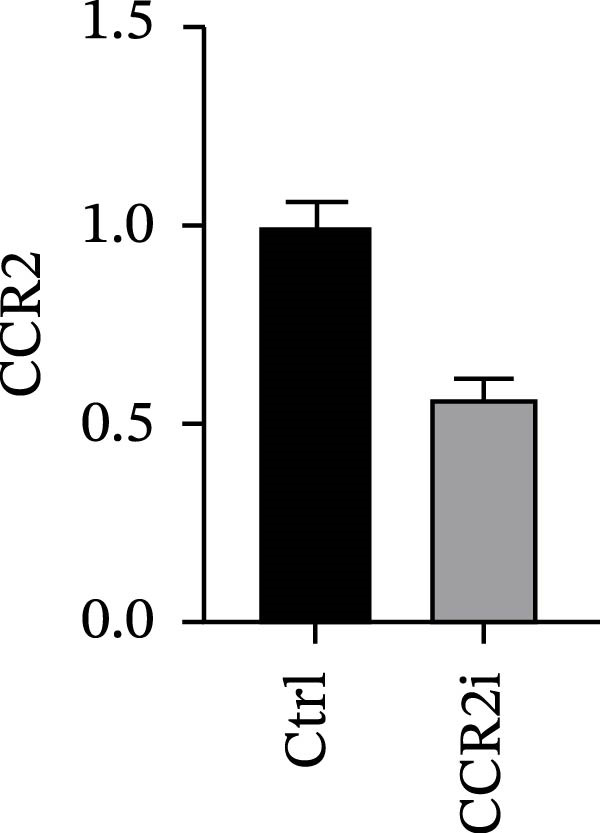
(J)

**Figure:**
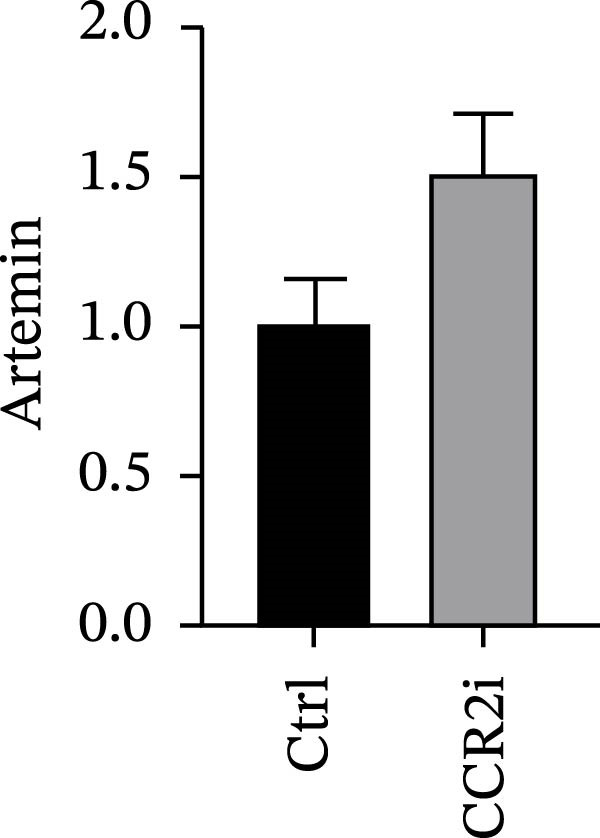
(K)

**Figure:**
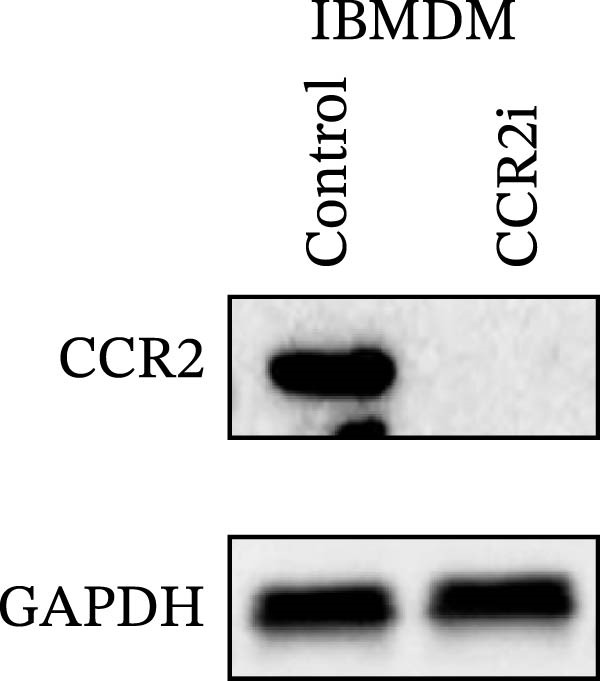
(L)

**Figure:**
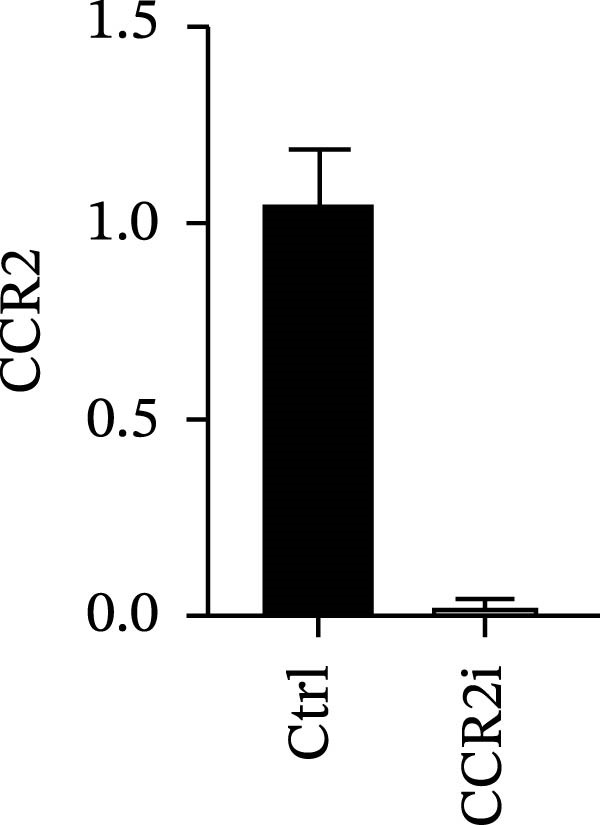
(M)

**Figure:**
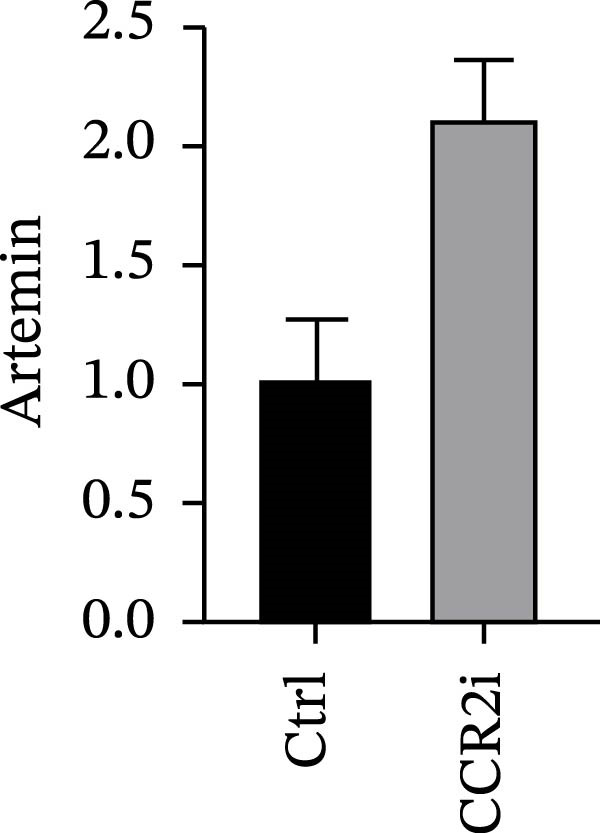
(N)

### 3.5. Biological Pathway and Gene‐Set Analyses

To contextualize the immune traits within broader biological networks, we next performed gene‐ and pathway‐level analyses. Gene‐level association (MAGMA) across the eight causal immune traits identified 129 pleiotropic genes (Bonferroni threshold *p*  < 3 × 10^−6^; Supporting Information 2: Table S10), and cross‐database annotation including OMIM/UniProt/DrugBank mapping is provided in Supporting Information 2: Table S11. Tissue‐expression enrichment implicated whole blood and spleen (Figure 6A,B; Supporting Information 2: Tables S12,S13). Integrated pathway analyses (KEGG, WP, and GO) highlighted external side of plasma membrane (GO:0009897), 16p11.2 distal deletion syndrome (WP4950), and IgG binding (GO:0019864) (Figure 6C; Supporting Information 2: Table S14). Protein–protein interaction (PPI) modules emphasized chemokine receptors (*CCR1*/*CCR2*/*CCR3*/*CCR5*) and Fc*γ*–receptor nodes (FCGR2A/FCGR2B) (Figure 6D), consistent with membrane‐proximal immune signaling relevant to myeloid leukemogenesis.

Figure 6Identification and analysis of pleiotropic genes for causal immune cell traits in hematologic malignancies. (A) Expression values of 129 pleiotropic genes identified through MAGMA gene analysis across 54 different tissues. (B) Tissue‐specific enrichment analysis demonstrating significant enrichment of pleiotropic genes in whole blood and spleen tissues. (C) Pathway analyses (KEGG, Wiki, and GO) conducted post gene mapping integration with MAGMA gene analysis, showcasing relevant biological processes and pathways. (D) Protein–protein interaction (PPI) networks of pleiotropic genes.

**Figure:**
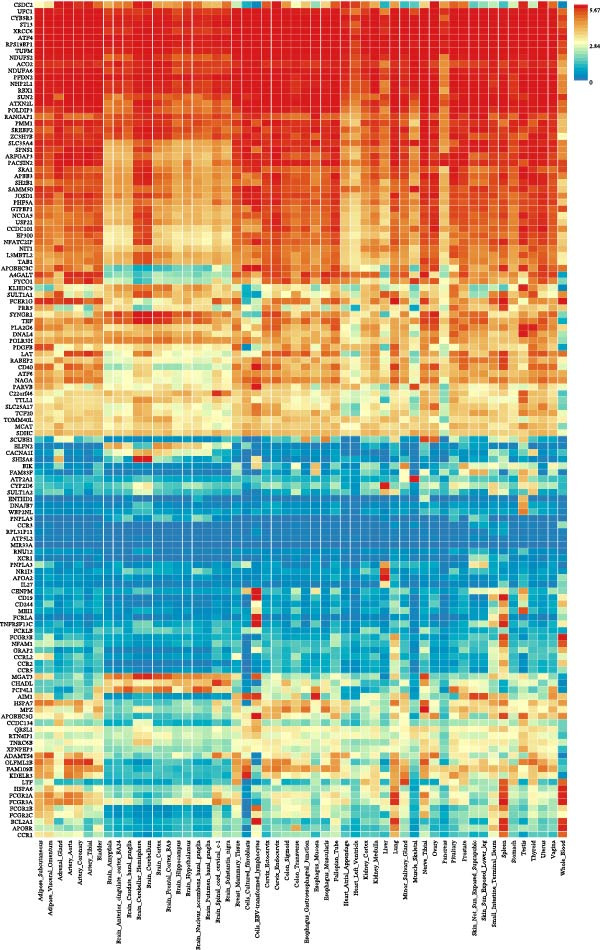
(A)

**Figure:**
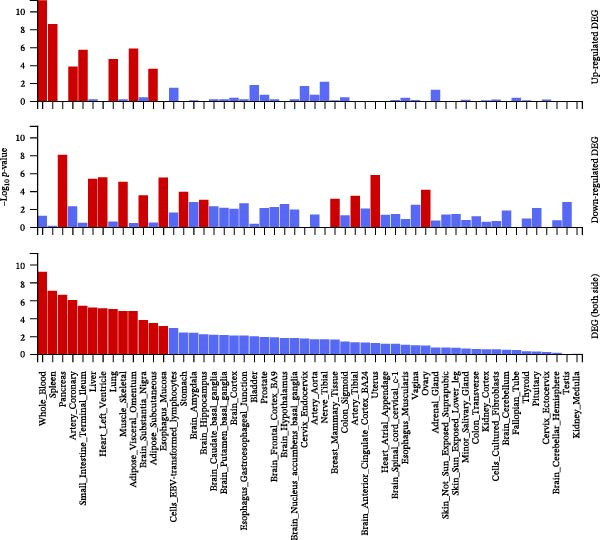
(B)

**Figure:**
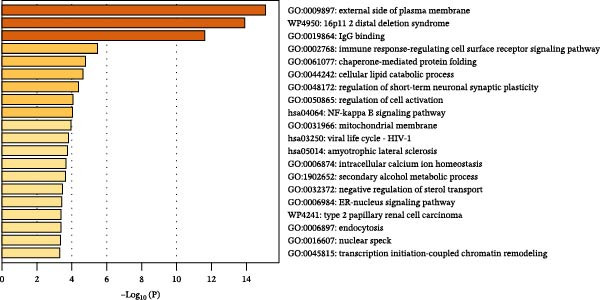
(C)

**Figure:**
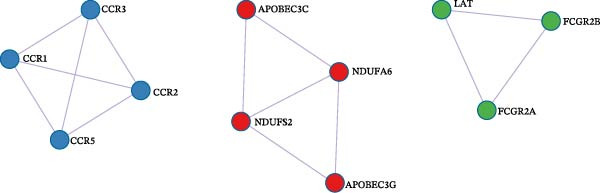
(D)

## 4. Discussion

In this study, we conducted a comprehensive, multistage investigation to uncover immune cell phenotypes that causally influence hematologic malignancies, using an integrated approach combining MR, mediation analysis, transcriptomic annotation, GRS–based validation, and molecular experiments. We first performed a two‐sample MR analysis involving 731 immune cell traits across 12 hematologic cancer types, identifying eight immune cell phenotypes significantly associated with disease risk. These associations were confirmed through sensitivity analyses and further refined using MVMR, revealing seven independent causal immune cell phenotypes. Mediation analysis further revealed four immune‐inflammation‐cancer axes—*CD40L*, *IL-33*, and notably *ARTN*—highlighting distinct cytokine pathways linking immune dysregulation to malignancy. External validation using trait‐specific GRSs in the UKB cohort confirmed consistent risk directions, most prominently showing that *CCR2* expression on *CD62L* ^+^ myeloid DCs was inversely associated with AML risk. Guided by these convergent genetic and epidemiologic findings, we provided functional support for the *CCR2-ARTN* regulatory axis in myeloid models relevant to AML. While *CCR2* signaling is conserved across the monocyte‐DC continuum, our assays used monocyte/macrophage models rather than primary *CD62L* ^+^ mDCs; we therefore interpret the data as directional evidence.

The established roles of *BAFF*, *CD19*, and *CD27* in hematologic malignancies were recapitulated by our MR analysis, serving as validation of the analytical framework. Consistent with previous reports, BAFF and its receptors (*BAFF-R*, *TACI*, and *BCMA*) are expressed in AML and other hematologic cancers, where their dysregulation contributes to apoptosis resistance and therapeutic response heterogeneity [32–36]. *CD19*, a well‐recognized B‐lineage marker, remains an effective immunotherapeutic target in B cell malignancies but shows limited applicability in AML except for rare cases with aberrant expression [37–41]. Likewise, *CD27* and its ligand *CD70* act as costimulatory molecules promoting lymphocyte activation, proliferation, and antitumor immunity, with elevated serum *CD27* levels reflecting tumor burden and therapeutic responsiveness [42–46]. In addition to confirming established immune regulators, our study identified new roles for *CD24*, *CD38*, and *HLA-DR* in hematologic malignancies, highlighting potential therapeutic avenues. *CD24* overexpression promotes tumor immune evasion by inhibiting macrophage phagocytosis, and its blockade has been shown to restore antitumor activity in B cell malignancies [47, 48]. *CD38*, a multifunctional signaling molecule, shapes BCR organization and can contribute to tumor progression [49–51], whereas HLA‐DR^+^ NK cells display distinct, functionally active states with enhanced effector functions, including cytokine production and T cell modulation [52–54]. Coexpression of HLA‐DR and *CD38* on lymphocytes correlates with increased cytotoxicity against HL cells [55, 56]. In addition, HLA‐DR–directed antibodies demonstrate antitumor activity in preclinical lymphoma models [57].

The key finding of this study lies in identifying *CCR2-ARTN* as a previously unrecognized immunoregulatory and metabolic signaling axis. Independent genomic and transcriptomic evidence positions *CCR2* as a druggable and monitorable node across hematologic settings. Chemokine‐gene prognostic models retain *CCR2* with characteristic immune‐cell correlates [58]; large‐scale GPCR transcriptomics in AML identify *CCR2* among upregulated, potentially druggable receptors across genetic subgroups [59]; and immune‐trait MR in B cell malignancies independently prioritizes *CCR2* as a candidate target, supporting the portability of human‐genetic target discovery [60]. Translationally, *CCR2* mRNA behaves as a robust, dose‐responsive pharmacodynamic biomarker with BRD4/BET inhibition (AZD5153) in blood and tumor, enabling on‐pathway monitoring in early trials [59]. While these studies do not specify causal direction, they corroborate the actionability and measurability of *CCR2*. Contextual literature further situates *CCR2* within an immunosuppressive chemokine hub. S100A4 amplifies myeloid‐derived suppressor cell (MDSC) programs via *GP130*/*JAK2*/*STAT3* and upregulates *CD14*/*CCR2*/*CCL2*, linking high *S100A4* to adverse prognosis and therapy insensitivity [61]. Oxidative‐stress signatures centered on *GPX1* correlate with MDSC infiltration, immune checkpoint engagement, and *CCR2* expression; *GPX1* knockdown reduces AML viability and *CCR2* levels [62]. Conversely, in *MYC*/*BCL2* double‐expressor DLBCL, *CCL2*/*CCR2* elevation accompanies M2 macrophage polarization, reduced T cell infiltration, and poor prognosis, illustrating disease‐ and lineage‐dependent *CCR2* biology [63]. An important nuance is compartment specificity. Studies focused on tumor cells show that blocking *CCR2* can sensitize AML cells to MEK inhibition and reduce prosurvival signaling, nominating *CCR2* inhibition as a therapeutic chemosensitizer in that setting [64]. By contrast, other work indicates that while *CCR2* supports transmigration and modest proliferation, it does not generally confer chemotherapy protection in AML, underlining that the axis is not a universal resistance pathway [65]. Our results resolve these threads by showing that, at the level of immune etiology, higher *CCR2* tone in *CD62L* ^+^ mDCs is associated with lower *ARTN* and lower AML risk. We therefore view *CCR2* as a direction‐dependent, compartment‐specific target: Enhancing *CCR2* function in immune cells may be protective, whereas inhibiting *CCR2* within leukemic cells may improve therapeutic response—two strategies that are not contradictory but complementary when deployed with cellular specificity.

*ARTN* emerges from our pipeline as the unique, coherent mediator linking immune‐cell traits to AML risk. Beyond mediation MR and myeloid perturbation data here, independent evidence shows *ARTN* drives oncogenic programs via RET‐AKT/mTOR and *STAT3* signaling and associates with adverse outcomes across solid tumors [66–72. Crucially, the hematopoietic system itself can be a systemic source of *ARTN*: Splenic CD45^-^Ter119^+^ erythroid‐lineage cells expand in myeloproliferative disease and secrete *ARTN*; arachidonic‐acid signaling enhances their myeloid differentiation potential [73]. The metabolites of arachidonic acid play a significant role in the blood system and immune responses, enhancing the differentiation potential of myeloid cells [74]. A recent review highlights tumor‐conditioned erythroid progenitors as immunosuppressive actors that secrete *ARTN* to promote tumor progression, reinforcing a concept of *ARTN* as a circulating effector that shapes the tumor‐immune set point [75]. These data provide a mechanistic substrate for our observation that *ARTN* partially mediates the protective association of *CCR2* ^+^ *CD62L* ^+^ mDCs with AML risk and motivate prioritization of RET/*ARTN*–axis interception alongside *CCR2*–directed strategies.

Beyond the expression‐level validation, we further explored the signaling implications of the *CCR2*‐*ARTN* axis through large‐scale proteomic correlation analyses. Using the UKB Olink inflammatory panel, *ARTN* was found to be coregulated with a cluster of immune‐metabolic proteins, including *CLEC6A*, *SIGLEC6*, *NPC2*, and *MTHFD2* (FDR < 0.05). These proteins map to glycan sensing and myeloid activation (*CLEC6A*/*SIGLEC6*), lysosomal‐cholesterol handling (*NPC2*), and mitochondrial one‐carbon/redox control (MTHFD2), functions that are compatible with downstream *AKT-STAT3* and oxidative‐stress signaling. Notably, *CCR2* pathway upregulation has been linked to *STAT3* activation in AML [61], while elevated *GPX1* expression—an oxidative‐stress regulator—has been shown to enhance *CCR2* levels and promote immunosuppressive MDSC accumulation in AML [62]. Complementary pathway enrichment revealed signals in external plasma‐membrane compartments, IgG binding/Fc*γ*R engagement, and a 16p11.2 locus involving *MAPK* and drug‐response regulation [76–78], findings that are broadly compatible with a membrane‐centric signaling model and the tractability of antibody‐ or receptor‐targeted strategies [79, 80].

Together, these multilayered data provide orthogonal support for a *CCR2-ARTN* pathway in AML; our myeloid assays further map *CCR2-ARTN* coupling and generate directional hypotheses for dendritic compartments. Because our assays used monocyte/macrophage models rather than primary *CD62L* ^+^ myeloid DCs, we interpret the experimental evidence as directional rather than definitive, pending orthogonal validation in primary *CD62L* ^+^ mDCs. Although our experimental validation primarily focused on *ARTN* expression changes following *CCR2* perturbation, the proteomic correlations and enrichment results extend the mechanistic interpretation toward downstream signaling and stress‐response contexts. Future studies should directly validate this pathway in primary *CD62L* ^+^ myeloid DCs or mDC–like systems, incorporating *CCR2* rescue or *ARTN* neutralization and assessing *ARTN*–dependent AKT/STAT3 activation and cellular phenotypes such as proliferation, apoptosis, and migration to refine the mechanistic framework and evaluate the therapeutic potential of targeting the *CCR2-ARTN* axis in AML.

## 5. Limitation

This study has several limitations. First, although we implemented extensive sensitivity analyses and prefiltered instruments using the GWAS Catalog to limit horizontal pleiotropy, residual pleiotropy cannot be fully excluded. Instruments were selected at *p*  < 1 × 10^−5^ from immune‐trait GWASs of modest sample size; despite F‐statistics > 10, weak‐instrument bias remains a theoretical concern. Second, the mediation framework relied on pQTLs from a targeted inflammatory panel (91 proteins), which may miss intracellular or microenvironmental mediators; protein quantification (Olink NPX) is platform‐specific, and pQTL architecture may introduce heterogeneity across datasets. Variant‐level colocalization for exposure‐mediator‐outcome was not performed and will be an important extension to strengthen causal chains. Third, outcome definitions were harmonized across UKB and FinnGen, but ascertainment and coding differences could introduce phenotype heterogeneity. Fourth, GRS validation was conducted in predominantly European‐ancestry participants; transferability to other ancestries is unknown. Fifth, our functional assays used human THP‐1 and murine IBMDM monocyte/macrophage models rather than primary *CD62L* ^+^ myeloid DCs and focused on *ARTN* transcript changes; we did not assay downstream signaling, cellular phenotypes (proliferation, apoptosis, and migration), rescue experiments (*CCR2* reexpression or *ARTN* blockade), or DC‐leukemic coculture systems. We therefore view these data as directional and plan mDC–focused validation (e.g., primary *CD62L* ^+^ mDCs, MUTZ‐3, or moDCs from PBMCs) with pathway and phenotype readouts. Note on directionality: Mediation leverages pQTLs for circulating *ARTN* protein, whereas our assays measured *ARTN* transcripts in monocyte/macrophage models; readout/compartment differences and the conditional nature of the mediator path can affect path signs. Accordingly, we emphasize the net IDE and defer directional claims in *CD62L* ^+^ mDCs to future work. Sixth, the *ARTN*–centered proteomic correlations (*CLEC6A*, *SIGLEC6*, *NPC2*, and *MTHFD2*) are associative; replication in independent cohorts and perturbational proteomics will be required. Finally, pathway enrichments provide biological context but are agnostic to directionality and susceptible to category granularity; we interpret them as supportive rather than definitive.

## 6. Conclusions

By integrating MR, mediation analysis, pathway annotation, GRS validation, and targeted cell assays, we delineate an immune‐inflammation pathway in which *CCR2* on *CD62L* ^+^ myeloid DCs is inversely associated with AML risk, with *ARTN* contributing as a downstream mediator. *CCR2* perturbation in monocyte/macrophage systems was accompanied by changes in *ARTN* transcript levels, providing functional support for *CCR2-ARTN* coupling, although sufficiency and in vivo relevance remain to be established; direct validation in primary *CD62L* ^+^ dendritic compartments is a priority. These data are consistent with *CCR2* signaling in immune cells helping maintain an antileukemic milieu, while *ARTN* links immune variation to survival pathways in myeloid contexts. Two complementary, hypothesis‐generating therapeutic avenues merit evaluation in preclinical models: enhancing *CCR2* function in immune compartments and intercepting *RET/ARTN* signaling where active. *CCR2* and circulating *ARTN* may serve as exploratory pharmacodynamic readouts in early studies. All causal inferences rest on MR assumptions and should be confirmed by statistical colocalization, mechanistic assays, and in vivo models.

**Nomenclature**
ADCC: Antibody‐dependent cellular cytotoxicity
AML: Acute myeloid leukemia
APCs: Antigen‐presenting cells
ARTN: Artemin
BCR: B cell receptor
CCR2i: CCR2 inhibitor
CD2: Cluster of differentiation 2
CI: Confidence interval
CLL: Chronic lymphocytic leukemia
cHL: Classical Hodgkin lymphoma
CML: Chronic myeloid leukemia
DLBCL: Diffuse large B cell lymphoma
ECL: Enhanced chemiluminescence
Egger: MR‐Egger
FBS: Fetal bovine serum
FL: Follicular lymphoma
FUMA: Functional mapping and annotation
GO: Gene Ontology
GRS: Genetic risk score
HL: Hodgkin lymphoma
HRS: Hodgkin and Reed–Sternberg
IBMDM: Immortalized bone marrow‐derived macrophage
IDE: Indirect effect
IV: Instrumental variable
IVW: Inverse‐variance weighted
KEGG: Kyoto Encyclopedia of Genes and Genomes
KO: Knockout
LL: Lymphoblastic lymphoma
LOO: Leave‐one‐out
MDSC: Myeloid‐derived suppressor cell
ML: Myeloid leukemia
MM: Multiple myeloma
MR: Mendelian randomization
MVMR: Multivariable Mendelian randomization
NFL: Nonfollicular lymphoma
NHL: Non‐Hodgkin lymphoma
OR: Odds ratio
PVDF: Polyvinylidene difluoride
qRT‐PCR: Quantitative real‐time polymerase chain reaction
RS: Reed–Sternberg
SE: Standard error
siRNA: Small interfering RNA
SNP: Single nucleotide polymorphism
TBST: Tris‐buffered saline with Tween 20
THP‐1: Human monocytic leukemia cell line
TNKL: T/NK cell lymphoma
UKB: UK Biobank
WP: WikiPathways.

## Author Contributions

**Yi Jin and Hui-Min Lu**: conceptualization, methodology, validation, formal analysis, investigation, data curation, writing – original draft, visualization. **Xing-Hao Yu, Ming-** **Zhu Su, and Jun Li**: validation, formal analysis, investigation, writing – original draft. **Xiao-Min Li**: conceptualization, methodology, visualization, supervision, project administration. **Jian-Hua Jin, Li-Ting Zhang, and Yue Wang:** conceptualization, methodology, writing – review and editing, visualization, supervision, project administration, funding acquisition.

## Funding

This work was supported by the funds of Changzhou Sci & Tech Program (CJ20200004) to Jian‐Hua Jin; the funds of Changzhou Sci & Tech Program (CJ20230007) and Medical Education Collaborative Innovation Fund of Jiangsu University (JDY2023017) to Yue Wang; the Research project on high quality development of Hospital pharmacy, National Institute of Hospital Administration, NHC, China (NIHAYSZX2549), the Changzhou Young Scientific and Technological Talent Support Program (CZTJ‐2025‐32), the Jiangsu Province Young Scientific and Technological Talent Support Program (JSTJ‐2025‐799), the Open Funding Project of the Jiangsu Provincial Key Laboratory of Anesthesiology, Xuzhou Medical University (XZSYSKF2023011), the Scientific Research Program of the Jiangsu Pharmaceutical Association (202495005), and the Changzhou Sci&Tech Program (CJ20242012) to Yi Jin; the Hospital Pharmacy Management Research Special Project, Jiangsu Hospital Association (JSYGY‐2‐2024‐YS26) to Xiao‐Min Li; the Noncommunicable Chronic Diseases‐National Science and Technology Major Project (2024ZD0534700), the Suzhou Science and Technology Program Project (QNXM2024010), and the Jiangsu Provincial Health Commission Project (MQ2024022) to Xing‐Hao Yu.

## Ethics Statement

This study did not involve any experiments with human or animal subjects directly; therefore, ethical approval was not required. The analysis was based on publicly available, anonymized datasets.

## Consent

The authors have nothing to report.

## Conflicts of Interest

The authors declare no conflicts of interest.

## Supporting Information

Additional supporting information can be found online in the Supporting Information section.

## Supporting information


**Supporting Information 1** Supporting Information File 1: STROBE‐MR checklist.

**Supporting Information 2** Supporting Information Tables: Tables S1–S14. Table S1: Preanalysis GWAS Catalog screen for horizontal pleiotropy: instrumental SNPs flagged and excluded (*p* < 5 × 10^−8^) with biologically related traits to the outcomes. Table S2: Significant instrumental variables identified from immune cell phenotypes. Table S3: MR results for immune cell phenotypes associated with hematologic malignancies. Table S4: Sensitivity analyses for MR results of immune cell phenotypes. Table S5: MR–PRESSO analysis results for significant immune cell phenotypes in hematologic malignancies. Table S6: Sensitivity analyses for MR results of immune cell phenotypes after removing pleiotropic SNPs. Table S7: Reverse MR analysis results for immune cell phenotypes and hematologic malignancies. Table S8: Two‐step mediation MR of circulating inflammatory proteins for MR–significant immune trait–malignancy pairs. Reported are the path coefficients *β*a (exposure→protein) and *β*b (protein→outcome conditional on exposure) and the indirect effect (IDE = *β*a × *β*b) with bootstrap 95% CIs. Table S9: Raw Cq values from qRT‐PCR experiments in IBMDM and THP‐1 cells under CCR2 knockdown or pharmacological inhibition. Table S10: MAGMA gene‐level association results for pleiotropic genes across causal immune traits. Table S11: Functional annotation and druggability mapping of pleiotropic genes. Table S12: Expression values of pleiotropic genes across 54 different tissues.

**Supporting Information 3** Supporting Information Figures: Figures S1–S3. Figure S1: The scatter plots for the eight significant associations between immune cell phenotypes and hematologic malignancies identified in the MR analysis. Figure S2: The LOO sensitivity analysis results for the eight significant immune cell phenotypes from the MR analysis. The LOO plots display the *p* values of causal effects when each single SNP is excluded individually, indicating the robustness of the associations between immune cell phenotypes and hematologic malignancies. Figure S3: CCR2 knockdown or pharmacological inhibition led to decreased expression of IL‐33 and CD40 in both IBMDM and THP‐1 cells. (A, B) mRNA levels of IL‐33 and CD40 in IBMDM cells following CCR2 knockdown with two independent siRNAs (KO‐1 and KO‐2) compared to control. (C, D) Expression of IL‐33 and CD40 in THP‐1 cells after CCR2 knockdown (KO‐1). (E, F) Expression of IL‐33 and CD40 in THP‐1 cells treated with CCR2 antagonist 4 hydrochloride (10 *μ*M and 24 h) compared to vehicle control.

## Data Availability

The original contributions presented in the study are included in the article and *Supporting Information*. Further inquiries can be directed to the corresponding author. GWAS summary data are available in public, open access repositories corresponding to the original studies (e.g., GWAS Catalog and FinnGen). The individual‐level genetic and phenotype data require permission from the UK Biobank (Applications 76875 and 41542; https://www.ukbiobank.ac.uk/).
