# Supplementary material for: Convergent Multistage Evidence Implicates the CCR2–Artemin Immune–Inflammation Axis in Acute Myeloid Leukemia
Source: Mediators Inflamm. 2026 Jan 31;2026:2476470. doi: 10.1155/mi/2476470 (PMC12860421; doi:10.1155/mi/2476470)
Supplement: Supplementary file 1 — Supporting Information 1 Supporting Information File 1: STROBE‐MR checklist. [file MI-2026-2476470-s001.docx]

**STROBE-MR checklist of recommended items to address in reports of Mendelian randomization studies**^1^ ^2^

| **Item No.** | **Section** | **Checklist item** | **Page No.** | **Relevant text from manuscript** |
| --- | --- | --- | --- | --- |
| 1 | **TITLE and ABSTRACT** | Indicate Mendelian randomization (MR) as the study’s design in the title and/or the abstract if that is a main purpose of the study | Page 1-2 | Title: The phrase “causal effects” indicates the use of a Mendelian randomization (MR) approach to infer causality. Abstract: “We conducted a two-sample MR analysis to identify associations between immune cell phenotypes and twelve hematologic malignancies.” |
|  | **INTRODUCTION** |  |  |  |
| 2 | **Background** | Explain the scientific background and rationale for the reported study. What is the exposure? Is a potential causal relationship between exposure and outcome plausible? Justify why MR is a helpful method to address the study question | Page 2-3 | In the introduction, the manuscript explains the scientific background by describing the heterogeneity of hematologic malignancies and the essential role of the immune system in the progression and development of these cancers. Specific immune cell phenotypes have been linked to either promoting or inhibiting tumor development, with the manuscript highlighting examples such as APCs, BCR signaling, IL-6, and IL-7, illustrating how these immune cells and circulating inflammatory proteins shape the tumor microenvironment. The introduction justifies the use of Mendelian randomization (MR) to address the causal relationships between immune cell phenotypes, circulating inflammatory proteins, and hematologic malignancies. The rationale is to reduce confounding factors and reverse causation, which are limitations in observational studies, making MR a helpful method for providing more reliable causal inferences. |
| 3 | **Objectives** | State specific objectives clearly, including pre-specified causal hypotheses (if any). State that MR is a method that, under specific assumptions, intends to estimate causal effects | Page 2-3 | The manuscript clearly states that the objective of the study is to use Mendelian randomization to investigate the causal associations between various immune cell populations and twelve hematologic malignancies. It further aims to explore the mediating role of circulating inflammatory proteins in these associations. The MR approach, under the assumptions of genetic variants as instrumental variables, is explicitly mentioned as a method to minimize confounding and reverse causation, allowing for a more accurate estimation of causal effects. The objective is to identify novel targets for early intervention and develop improved prevention and treatment strategies. |
|  | **METHODS** |  |  |  |
| 4 | **Study design and data sources** | Present key elements of the study design early in the article. Consider including a table listing sources of data for all phases of the study. For each data source contributing to the analysis, describe the following: |  |  |
|  | a) | Setting: Describe the study design and the underlying population, if possible. Describe the setting, locations, and relevant dates, including periods of recruitment, exposure, follow-up, and data collection, when available. | Page 4 | Line 95-102 |
|  | b) | Participants: Give the eligibility criteria, and the sources and methods of selection of participants. Report the sample size, and whether any power or sample size calculations were carried out prior to the main analysis | Page 4-5 | Line 103-127 |
|  | c) | Describe measurement, quality control and selection of genetic variants | Page 5 | Line 128-135 |
|  | d) | For each exposure, outcome, and other relevant variables, describe methods of assessment and diagnostic criteria for diseases | Page 5-6 | Line 136-148 |
|  | e) | Provide details of ethics committee approval and participant informed consent, if relevant | Page 6 | Line 149-150 |
| 5 | **Assumptions** | Explicitly state the three core IV assumptions for the main analysis (relevance, independence and exclusion restriction) as well assumptions for any additional or sensitivity analysis | Page 5 | Line140-146 |
| 6 | **Statistical methods: main analysis** | Describe statistical methods and statistics used |  |  |
|  | a) | Describe how quantitative variables were handled in the analyses (i.e., scale, units, model) | Page 5 | Line 129-135 |
|  | b) | Describe how genetic variants were handled in the analyses and, if applicable, how their weights were selected | Page 5 | Line 129-135 |
|  | c) | Describe the MR estimator (e.g. two-stage least squares, Wald ratio) and related statistics. Detail the included covariates and, in case of two-sample MR, whether the same covariate set was used for adjustment in the two samples | Page 5-6 | Line 140-146 |
|  | d) | Explain how missing data were addressed | Page 5 | Line 132-135 |
|  | e) | If applicable, indicate how multiple testing was addressed | Page 6 | Line 144-146 |
| 7 | **Assessment of assumptions** | Describe any methods or prior knowledge used to assess the assumptions or justify their validity | Page 6 | Line 152-161 |
| 8 | **Sensitivity analyses and additional analyses** | Describe any sensitivity analyses or additional analyses performed (e.g. comparison of effect estimates from different approaches, independent replication, bias analytic techniques, validation of instruments, simulations) | Page 6 | Line 152-161 |
| 9 | **Software and pre-registration** |  |  |  |
|  | a) | Name statistical software and package(s), including version and settings used | Page 6 | Line 147-150 |
|  | b) | State whether the study protocol and details were pre-registered (as well as when and where) |  | Not Applicable |
|  | **RESULTS** |  |  |  |
| 10 | **Descriptive data** |  |  |  |
|  | a) | Report the numbers of individuals at each stage of included studies and reasons for exclusion. Consider use of a flow diagram | Page 8 | Line 201-206 |
|  | b) | Report summary statistics for phenotypic exposure(s), outcome(s), and other relevant variables (e.g. means, SDs, proportions) | Page 8 | Line 201-203 |
|  | c) | If the data sources include meta-analyses of previous studies, provide the assessments of heterogeneity across these studies |  | Not Applicable |
|  | d) | For two-sample MR:  i.  Provide justification of the similarity of the genetic variant-exposure associations between the exposure and outcome samples  ii.  Provide information on the number of individuals who overlap between the exposure and outcome studies | Page 8 | Line 207-221 |
| 11 | **Main results** |  |  |  |
|  | a) | Report the associations between genetic variant and exposure, and between genetic variant and outcome, preferably on an interpretable scale | Page 8 | Line 207-221 |
|  | b) | Report MR estimates of the relationship between exposure and outcome, and the measures of uncertainty from the MR analysis, on an interpretable scale, such as odds ratio or relative risk per SD difference | Page 8 | Line 207-221 |
|  | c) | If relevant, consider translating estimates of relative risk into absolute risk for a meaningful time period |  | Not Applicable |
|  | d) | Consider plots to visualize results (e.g. forest plot, scatterplot of associations between genetic variants and outcome versus between genetic variants and exposure) | Page 8 | Line 222-229 |
| 12 | **Assessment of assumptions** |  |  |  |
|  | a) | Report the assessment of the validity of the assumptions | Page 8-9 | Line 222-236 |
|  | b) | Report any additional statistics (e.g., assessments of heterogeneity across genetic variants, such as *I^2^*, Q statistic or E-value) | Page 8-9 | Line 222-236 |
| 13 | **Sensitivity analyses and additional analyses** |  |  |  |
|  | a) | Report any sensitivity analyses to assess the robustness of the main results to violations of the assumptions | Page 8-9 | Line 222-236 |
|  | b) | Report results from other sensitivity analyses or additional analyses | Page 8-9 | Line 222-236 |
|  | c) | Report any assessment of direction of causal relationship (e.g., bidirectional MR) | Page 8-9 | Line 222-236 |
|  | d) | When relevant, report and compare with estimates from non-MR analyses |  | Not Applicable |
|  | e) | Consider additional plots to visualize results (e.g., leave-one-out analyses) | Page 8-9 | Line 222-236 |
|  | **DISCUSSION** |  |  |  |
| 14 | **Key results** | Summarize key results with reference to study objectives | Page 10 | Line 268-281 |
| 15 | **Limitations** | Discuss limitations of the study, taking into account the validity of the IV assumptions, other sources of potential bias, and imprecision. Discuss both direction and magnitude of any potential bias and any efforts to address them | Page 14-15 | Line 405-416 |
| 16 | **Interpretation** |  |  |  |
|  | a) | Meaning: Give a cautious overall interpretation of results in the context of their limitations and in comparison with other studies | Page 10-14 | Line 282-404 |
|  | b) | Mechanism: Discuss underlying biological mechanisms that could drive a potential causal relationship between the investigated exposure and the outcome, and whether the gene-environment equivalence assumption is reasonable. Use causal language carefully, clarifying that IV estimates may provide causal effects only under certain assumptions | Page 10-14 | Line 282-404 |
|  | c) | Clinical relevance: Discuss whether the results have clinical or public policy relevance, and to what extent they inform effect sizes of possible interventions | Page 10 | Line 279-281 |
| 17 | **Generalizability** | Discuss the generalizability of the study results (a) to other populations, (b) across other exposure periods/timings, and (c) across other levels of exposure | Page 14-15 | Line 405-416 |
|  | **OTHER INFORMATION** |  |  |  |
| 18 | **Funding** | Describe sources of funding and the role of funders in the present study and, if applicable, sources of funding for the databases and original study or studies on which the present study is based | Page 16 | Line 449-451 |
| 19 | **Data and data sharing** | Provide the data used to perform all analyses or report where and how the data can be accessed, and reference these sources in the article. Provide the statistical code needed to reproduce the results in the article, or report whether the code is publicly accessible and if so, where | Page 16 | Line 452-454 |
| 20 | **Conflicts of Interest** | All authors should declare all potential conflicts of interest | Page 16 | Line 463-465 |

This checklist is copyrighted by the Equator Network under the Creative Commons Attribution 3.0 Unported (CC BY 3.0) license.

1. Skrivankova VW, Richmond RC, Woolf BAR, Yarmolinsky J, Davies NM, Swanson SA, et al. Strengthening the Reporting of Observational Studies in Epidemiology using Mendelian Randomization (STROBE-MR) Statement. JAMA. 2021;under review.

2. Skrivankova VW, Richmond RC, Woolf BAR, Davies NM, Swanson SA, VanderWeele TJ, et al. Strengthening the Reporting of Observational Studies in Epidemiology using Mendelian Randomisation (STROBE-MR): Explanation and Elaboration. BMJ. 2021;375:n2233.
