# Supplementary material for: Convergent Multistage Evidence Implicates the CCR2–Artemin Immune–Inflammation Axis in Acute Myeloid Leukemia
Source: Mediators Inflamm. 2026 Jan 31;2026:2476470. doi: 10.1155/mi/2476470 (PMC12860421; doi:10.1155/mi/2476470)
Supplement: Supplementary file 3 — Supporting Information 3 Supporting Information Figures: Figures S1–S3. Figure S1: The scatter plots for the eight significant associations between immune cell phenotypes and hematologic malignancies identified in the MR analysis. Figure S2: The LOO sensitivity analysis results for the eight significant immune cell phenotypes from the MR analysis. The LOO plots display the p values of causal effects when each single SNP is excluded individually, indicating the robustness of the associations between immune cell phenotypes and hematologic malignancies. Figure S3: CCR2 knockdown or pharmacological inhibition led to decreased expression of IL‐33 and CD40 in both IBMDM and THP‐1 cells. (A, B) mRNA levels of IL‐33 and CD40 in IBMDM cells following CCR2 knockdown with two independent siRNAs (KO‐1 and KO‐2) compared to control. (C, D) Expression of IL‐33 and CD40 in THP‐1 cells after CCR2 knockdown (KO‐1). (E, F) Expression of IL‐33 and CD40 in THP‐1 cells treated with CCR2 antagonist 4 hydrochloride (10 μM and 24 h) compared to vehicle control. [file MI-2026-2476470-s002.docx]

Supplementary Figures

***Causal and Mediated Effects of Circulating Inflammatory Proteins in the Link Between Immune Cells and Hematologic Malignancies: Insights from Large-Scale Genetic Analysis***


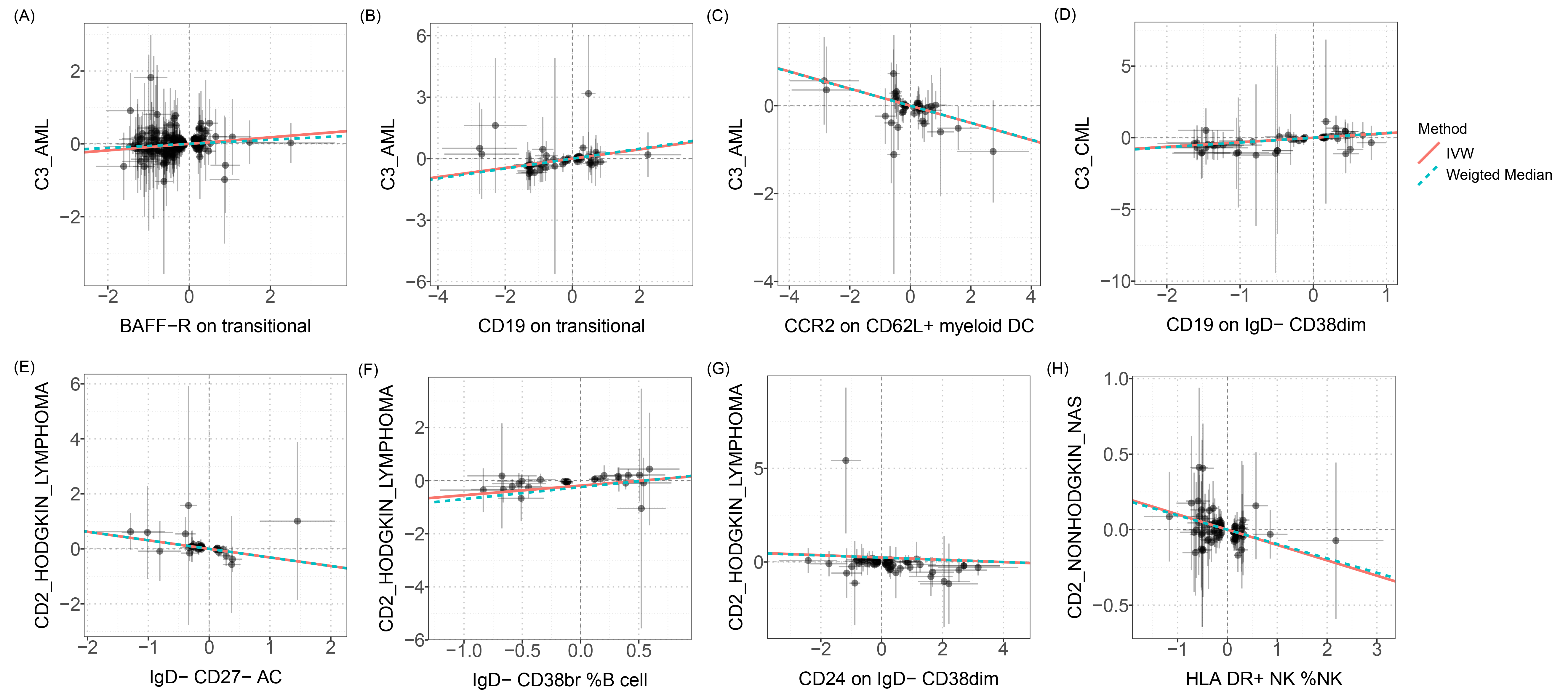


**Figure S1.** The scatter plots for the eight significant associations between immune cell phenotypes and hematologic malignancies identified in the MR analysis.


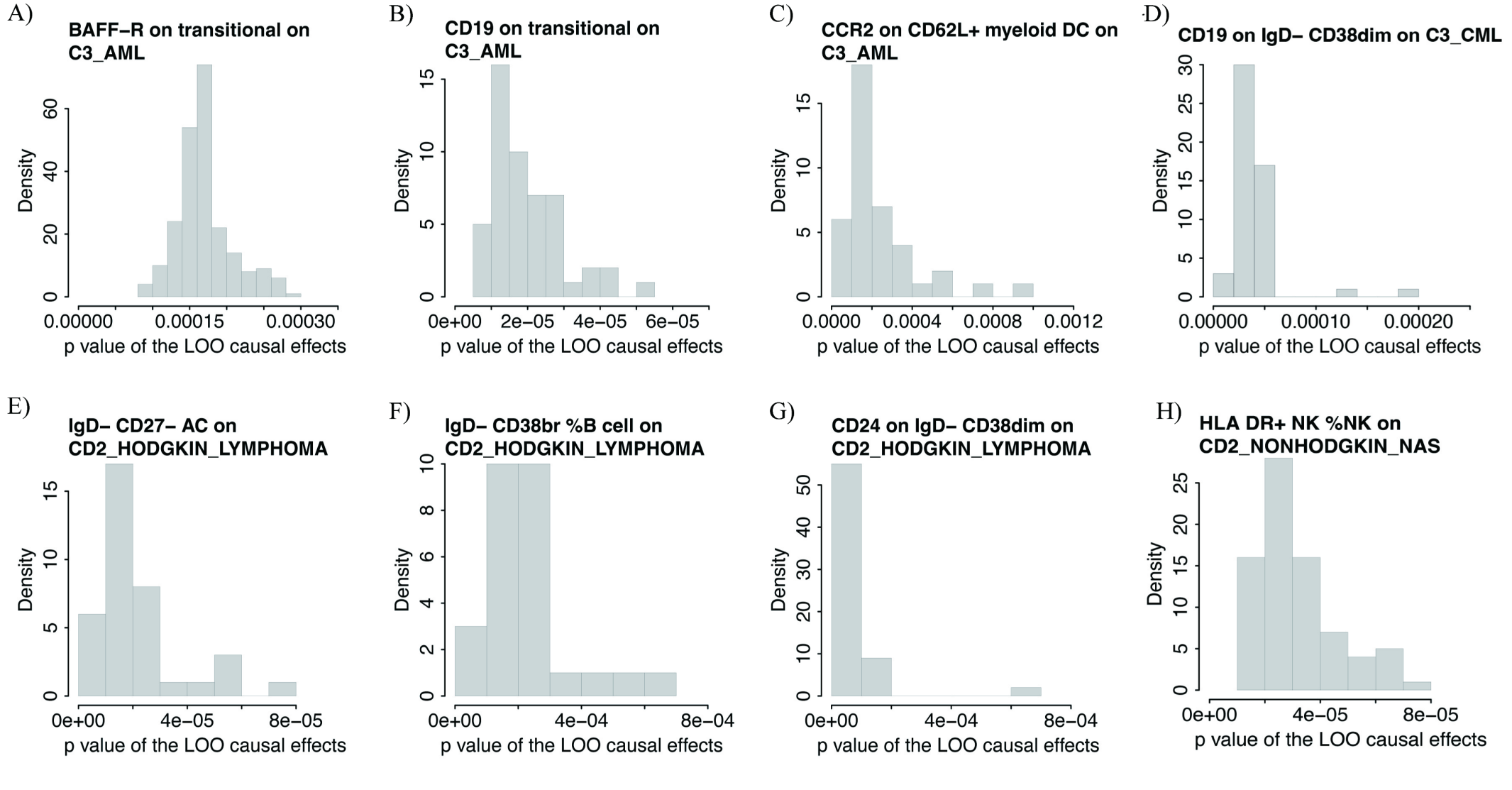


**Figure S2.** The LOO sensitivity analysis results for the eight significant immune cell phenotypes from the MR analysis. The LOO plots display the P values of causal effects when each single SNP is excluded individually, indicating the robustness of the associations between immune cell phenotypes and hematologic malignancies.


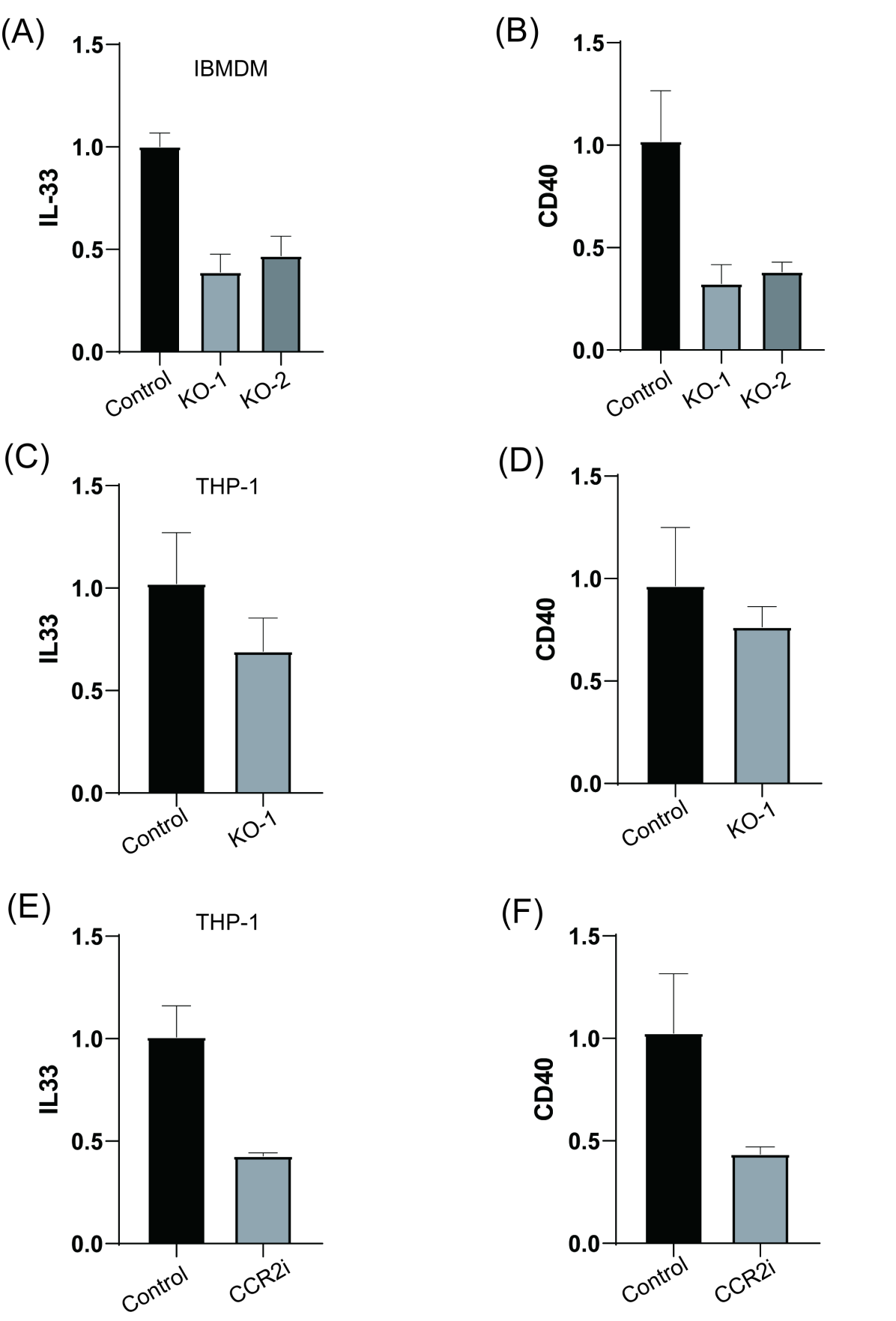


**Figure S3.** CCR2 knockdown or pharmacological inhibition led to decreased expression of IL-33 and CD40 in both IBMDM and THP-1 cells. (A-B) mRNA levels of IL-33 and CD40 in IBMDM cells following CCR2 knockdown with two independent siRNAs (KO-1, KO-2) compared to control. (C-D) Expression of IL-33 and CD40 in THP-1 cells after CCR2 knockdown (KO-1). (E-F) Expression of IL-33 and CD40 in THP-1 cells treated with CCR2 antagonist 4 hydrochloride (10 μM, 24 h) compared to vehicle control.
